# High Conservation of Translation-Enabling RNA Editing Sites in Hyper-editing Ferns Implies They Are Not Selectively Neutral

**DOI:** 10.1093/molbev/msaf241

**Published:** 2025-09-30

**Authors:** Farley M Kwok van der Giezen, Rose McDowell, Owen Duncan, Simon Zumkeller, Catherine Colas des Francs-Small, Ian Small

**Affiliations:** ARC Centre of Excellence in Plants for Space, School of Molecular Sciences, The University of Western Australia, Perth, WA 6009, Australia; School of Molecular Sciences, The University of Western Australia, Perth, WA 6009, Australia; Western Australian Proteomics Facility, School of Molecular Sciences, The University of Western Australia, Perth, WA 6009, Australia; Institute of Bio- and Geosciences, IBG-4: Bioinformatics, Forschungszentrum Jülich, Jülich D-52428, Germany; Cluster of Excellence on Plant Sciences (CEPLAS), Heinrich-Heine-Universität Düsseldorf, Düsseldorf 40225, Germany; School of Molecular Sciences, The University of Western Australia, Perth, WA 6009, Australia; ARC Centre of Excellence in Plants for Space, School of Molecular Sciences, The University of Western Australia, Perth, WA 6009, Australia

**Keywords:** RNA editing, ferns, *Salviniales*, constructive neutral evolution, chloroplast, mitochondria

## Abstract

RNA editing is essential for processing transcripts in plant chloroplasts and mitochondria. Levels of RNA editing vary between lineages, but some hornworts, lycophytes, and ferns have an extraordinary abundance of RNA editing. A feature of “hyper-editing” species is the prevalence of RNA editing events that promote translation by creating start codons or removing stop codons via C-to-U or U-to-C editing, respectively. These “translation-enabling” RNA editing events may play roles in regulating organelle gene expression. To investigate the importance of translation-enabling RNA editing events, we generated DNA and RNA sequence libraries for four *Salviniales* water ferns: *Azolla rubra*, *Azolla pinnata*, *Marsilea mutica*, and *Salvinia molesta*. We assembled chloroplast genomes, mitochondrial genes, and transcriptomes and identified RNA editing sites and candidate RNA editing factors. We reanalyzed sequence data of *Azolla filiculoides* and conducted a comparative analysis of RNA editing in chloroplasts and mitochondrial transcripts. Estimates of pyrimidine transition rates show that translation-enabling RNA editing sites are more conserved than other nonsynonymous editing sites, suggesting an emergent role in organelle gene expression that is not selectively neutral. This makes these events rare examples of RNA editing conferring adaptive advantage, in contrast to the prevailing view that RNA editing arises via constructive neutral evolutionary processes. Shotgun proteomics of *M. mutica* chloroplast thylakoid fractions verified the expected consequences of RNA editing on translation of chloroplast transcripts and implies that mechanisms exist to avoid translation of partially edited transcripts. Start codon editing may be one of those mechanisms.

## Graphical Abstract

**Graphical Abstract:**
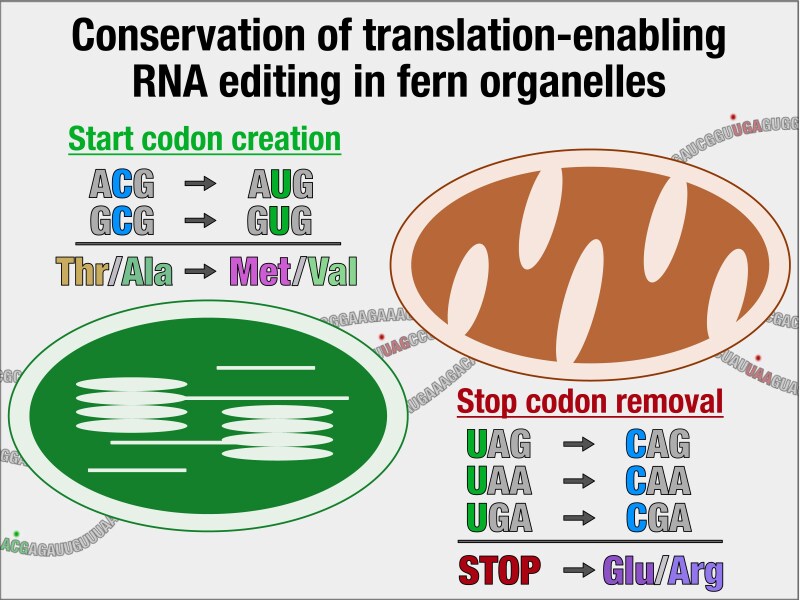

## Introduction

RNA editing is an essential posttranscriptional process in the expression of many chloroplast and mitochondrial genes. In terrestrial plants, RNA editing is most commonly present as deamination of cytidine to uridine (C-to-U) (Covello and Gray 1989; Hiesel et al. 1989; Gualberto et al. 1990; Chateigner-Boutin and Small 2010). Less common is the conversion of uridine to cytidine (U-to-C). U-to-C editing is confined to the chloroplast and mitochondrial transcripts of hornworts (Kugita et al. 2003; Gerke et al. 2020), lycophytes (Grewe et al. 2011; Oldenkott et al. 2014; Kwok van der Giezen et al. 2024), and ferns (Yoshinaga et al. 1996; Wolf et al. 2004; Guo et al. 2017; Fauskee et al. 2021). RNA editing plays diverse roles in chloroplast and mitochondrial transcript processing. It is present in tRNAs (Binder et al. 1994), rRNAs (Hecht et al. 2011), and introns and untranslated regions (Castandet et al. 2010; Oldenkott et al. 2014), but it is most abundant in the coding regions of mRNAs (Hecht et al. 2011). In protein-coding regions, both C-to-U and U-to-C RNA editing act to restore codons encoding evolutionarily conserved amino acids. In some cases, this has a significant and crucial impact on translation. C-to-U editing can create start codons and stop codons to allow proper initiation and termination (Neckermann et al. 1994; Wolf et al. 2004; Ichinose and Sugita 2016), and U-to-C editing can remove premature stop codons which are abundant as apparent nonsense mutations in the protein-coding genes of many hornwort, lycophyte, and fern chloroplasts and mitochondria (Steinhauser et al. 1999; Wolf et al. 2004; Grewe et al. 2011).

RNA editing is present in all lineages of terrestrial plants except for a single case of a secondary loss in the *Marchantiid* liverwort *Marchantia polymorpha* (Rüdinger et al. 2008). In seed plants, there is a trend of gradually decreasing levels of RNA editing (Freyer et al. 1997; Mower 2008; Fujii and Small 2011; Ishibashi et al. 2019), but in nonseed plants, there is remarkable variation in the abundance of RNA editing. In the moss *Physcomitrium patens*, there are only 13 RNA editing sites in the chloroplasts and mitochondria (Miyata and Sugita 2004; Rüdinger et al. 2009), while at the other end of the spectrum, the lycophyte *Selaginella uncinata* has more than 3,400 editing sites in its chloroplasts alone (Oldenkott et al. 2014). Here, we consider “hyper-editing” species to be those with over 1,000 editing events in either chloroplasts or mitochondria, or both. Such hyper-editing is also seen in species of hornworts such as *Anthoceros agrestis* (Guo et al. 2015, 2017; Knie et al. 2016; Li et al. 2018; Gerke et al. 2020) and in many eusporangiate and leptosporangiate ferns (Guo et al. 2015, 2017; Knie et al. 2016; Li et al. 2018; Zumkeller and Knoop 2023). The first fern nuclear genomes to be assembled were from the aquatic ferns *Azolla filiculoides* and *Salvinia cucullata*, where initial analysis of organelle RNA editing was estimated at over 1,700 and 1,200 sites, respectively (Li et al. 2018). Relative to seed plants, nonseed plants are understudied. However, their RNA editing patterns can provide valuable insights into how plant RNA editing has evolved.

The enzymes responsible for RNA editing in chloroplasts and mitochondria are the RNA binding protein family pentatricopeptide repeat (PPR) proteins. PPR proteins have undergone massive expansion in land plants (Gutmann et al. 2020). They have several roles in posttranscriptional processing of chloroplast and mitochondrial transcripts. P-class PPR proteins, named for their tandem repeats of the 35-amino acid P repeat, can act to bind UTRs of transcripts to stabilize and protect them from exonucleolytic digestion (Pfalz et al. 2009; Ruwe and Schmitz-Linneweber 2012; Zhelyazkova et al. 2012), or are required for RNA splicing (Schmitz-Linneweber et al. 2006; Aryamanesh et al. 2017; Lee et al. 2019), or promote RNA cleavage with implications for cytoplasmic male sterility (CMS) (Gobert et al. 2010; Binder et al. 2013; Zhou et al. 2017; Melonek et al. 2021; Huynh et al. 2023). Most PPR proteins involved in RNA editing are PLS-class PPR proteins, so named for repeating triplets of P (35 aa), L (35-36 aa) and S (31-32 aa) repeats (Lurin et al. 2004; Cheng et al. 2016). Many PLS-class PPR proteins encode an extended C-terminal cytidine deaminase-like “DYW” domain that catalyzes C-to-U deamination (Lurin et al. 2004; Salone et al. 2007; Boussardon et al. 2012; Oldenkott et al. 2019; Hayes and Santibanez 2020; Takenaka et al. 2021), or a “DYW:KP” domain which, despite its high degree of sequence similarity to the DYW domain, catalyzes U-to-C amination by an as-yet unknown mechanism (Gerke et al. 2020; Gutmann et al. 2020; Ichinose et al. 2022, 2025; Chun et al. 2025; Hayes et al. 2024). PPR proteins are one of the largest protein families in plants, accounting for nearly 10% of mRNA transcript diversity in some hyper-editing species (Gutmann et al. 2020). The abundance of PPR RNA editing factors correlates strongly with the abundance of RNA editing in hyper-editing species (Gutmann et al. 2020). Such heavy investment in organelle RNA processing would seem likely to result in a metabolic burden for hyper-editing plants; however, hyper-editing species within the Salviniaceae are among the fastest growing plants on Earth (Sale et al. 1985; Wagner 1997).

**Fig. 1.**
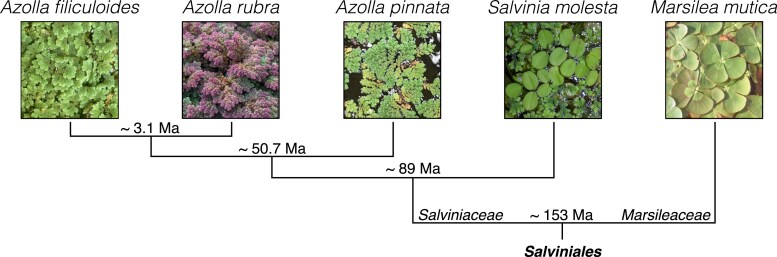
Dated phylogeny of *Salviniales* ferns. Divergence estimates based on Pryer et al. (2004), Metzgar et al. (2007), and Testo and Sundue (2016).

RNA editing has been proposed to have emerged and accelerated through a process of constructive neutral evolution (CNE) (Covello and Gray 1993; Gray 2012). This model posits that RNA editing confers a tolerance for deleterious DNA mutations, allowing such mutations to accumulate (Gray 2012). At first sight, the paired expansion of PLS-class PPR proteins and RNA editing sites within chloroplasts and mitochondria in hyper-editing species fits neatly in the model of CNE, where the rapid gain of new editing proteins confers tolerance to deleterious mutations which accumulate at a greater rate than can be fixed at the DNA level. This can act as a unidirectional evolutionary ratchet that results in gains of editing sites in hyper-editing species (Lukeš et al. 2011; Gray 2012). However, closer inspection reveals differences in conservation of editing sites, suggesting possible differences in mutation rates or selection pressure. C-to-U and U-to-C sites are conserved differently, and translation-enabling editing sites (those that create start codons and remove stop codons) have been observed to be retained at higher rates than other sites (Li et al. 2018; Fauskee et al. 2021, 2025). Empirical testing of the CNE hypothesis against alternative hypotheses has been limited by available sequence data for hyper-editing species. Using a set of *Salviniales* ferns *Azolla rubra*, *Azolla pinnata*, *Salvinia molesta*, and *Marsilea mutica* and the available data for *A. filiculoides* (Fig. 1) (Li et al. 2018), we assembled chloroplast genomes and mitochondrial genes and used deep RNA sequencing to characterize organelle “editomes.” Comparative analysis of these editomes reveals translation-enabling RNA editing sites are more conserved relative to other editing sites because reversional DNA mutations at these sites are rare. The implication is that these plants benefit from creating translatable mRNAs by RNA editing rather than having the translatable sequences encoded in their organelle genomes.

## Results

High coverage DNA- (20 M reads) and RNA-seq (110 M reads) datasets were generated for *A. rubra*, *A. pinnata*, *S. molesta*, and *M. mutica*. Chloroplast genomes and mitochondrial contigs containing all known genes were assembled for each species and were used as references for mapping RNA reads to detect RNA editing sites. Annotated gene models were greatly improved after manual curation of annotations to account for the creation of start and stop codons and removal of premature stop codons by RNA editing. The *A. filiculoides* data from Li et al. (2018) were reanalyzed using our RNA editing detection pipeline and annotation methods.

### Features of the *Salviniales* Plastomes

The plastomes of *A. rubra*, *A. pinnata*, *S. molesta*, and *M. mutica* have typical quadripartite structures containing a large single-copy (LSC) region and a small single-copy (SSC) region separated by two inverted repeat (IR) regions (Figures S1 to S4). *Salviniales* plastomes contain 87 unique protein-coding genes, except for *A. pinnata* in which the *rpl22* gene has lost both its start and stop codons, and there is no RNA editing that restores a functional coding sequence. The *Salviniales* plastomes encode just 27 unique tRNAs. They, like other leptosporangiate ferns *Adiantum capillus-veneris* (Wolf et al. 2003) and *Alsophila spinulosa* (Gao et al. 2009), lack a gene encoding tRNA^Lys^ which may be imported into their chloroplasts (Berrissou et al. 2024). Each plastome contains four duplicated rRNA genes. Also present in the *Salviniales* plastomes is the *ffs* gene encoding a noncoding RNA component of the chloroplast signal recognition particle (Träger et al. 2013) and *ycf94* which encodes a predicted transmembrane domain protein in ferns, lycophytes and some bryophytes (Song et al. 2018). The plastomes were examined for the presence of mobile open reading frames in fern organelles (Robison et al. 2018), but these appear to be absent in all four species. Features of the *Salviniales* plastomes and updated annotation features of the *A. filiculoides* chloroplast genome (MF177094) are summarized in Table 1.

**Table 1:** Summary of *Salviniales* chloroplast genome feature**s**

	*Azolla filiculoides* ^a^ (MF177094)	*Azolla rubra* (PQ616047)	*Azolla pinnata* (PQ616048)	*Salvinia molesta* (PQ616050)	*Marsilea mutica* (PQ616049)
Chloroplast genome size (bp)	147,665	147,792	146,849	149,624	154,342
LSC (bp)	83,453	83,434	83,062	83,400	85,091
SSC (bp)	22,168	22,320	21,545	24,288	22,433
IR (bp)	21,022	21,019	21,121	20,968	23,409
GC%	42.2	42.2	40.5	41.8	42.6
Number of genes^b^	119 (132)	119 (132)	118 (131)	119 (132)	119 (132)
Protein-coding	87 (91)	87 (91)	86 (90)	87 (91)	87 (91)
ffs ncRNA	1	1	1	1	1
tRNA	27 (32)	27 (32)	27 (32)	27 (32)	27 (32)
rRNA	4 (8)	4 (8)	4 (8)	4 (8)	4 (8)

Annotations for the *Salviniales* chloroplast genomes were manually curated to account for translation-enabling RNA editing events that create or remove start and stop codons. Reannotation MF177094 resulted in the addition of annotations for the *ycf94* gene and *ffs* noncoding RNA, modification of start codon coordinates for *psbC*, *rps11*, and *atpI* and modification of stop codon coordinates for *rpl20*, *petD*, and *rpoA*.

^a^Chloroplast content for *A. filiculoides* MF177094 is based on our reannotation with manual curation after reanalysis of RNA editing.

^b^Total gene counts including duplicates are indicated in parentheses.

### Assembly of *Salviniales* Mitochondrial Genes

Attempts to assemble complete mitochondrial genomes failed due to long (i.e. longer than the read length) regions of microsatellite-like tandem repeats (Figure S5). It is possible that the mitochondrial genomes of *Salviniales* are present as several chromosomes with multiple possible arrangements with dozens of recombination breakpoints, like the mtDNA of *Haplopteris ensiformis* (Zumkeller et al. 2023). Conserved RNA editing events occur mainly in protein-coding regions; therefore, we focused on generating short contigs which encompass the 36 mitochondrial protein-coding genes annotated in *A. filiculoides* (MN400566 to MN400574) (Feng and Wicke 2022).

### The Mitochondrial Gene Complement of *Salviniales*

**Fig. 2.**
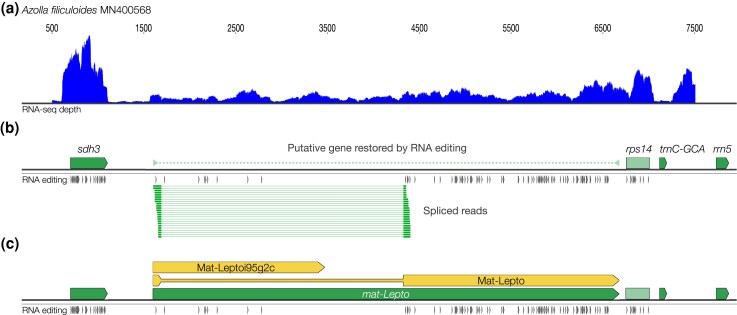
A novel maturase/reverse transcriptase-like gene identified in an unannotated region of the *A. filiculoides* mitochondrial genome contains two open reading frames. a) The RNA coverage of the *mat-Lepto* region within contig MN400568 is low but continuously above background levels. b) Transcripts between *sdh3* and *trnC-GCA* contain extensive RNA editing. Examination of this region revealed extensive RNA editing and evidence of RNA splicing to create two open reading frames. c) *mat-Lepto* encodes two proteins; Mat-Leptoi95g2c can be translated from the unspliced mRNA and resembles an intron maturase, so is likely involved in splicing of the host intron to allow translation of Mat-Lepto, which also resembles an intron maturase, that presumably acts on one or more other introns in the mitochondrial transcriptome. The splice junction was verified from RNA reads mapping to the MN400568 mitochondrial DNA sequence.

The *A. rubra*, *A. pinnata*, *S. molesta*, and *M. mutica* mitochondrial gene complements match that of *A. filiculoides* (Feng and Wicke 2022). Genes encoding subunits of respiratory complexes I to V are present (*atp*, *cob*, *cox*, *nad* genes), including *sdh3* and *sdh4* that encode subunits of complex II. Leptosporangiate ferns, including the *Salviniales* ferns and the fern *H. ensiformis*, lack *ccm* genes for cytochrome *c* maturation (*ccmB*, *ccmC*, *ccmF*) (Zumkeller et al. 2023). The *Salviniales* do not show any indication of encoding the MatR intron maturase. In *H. ensiformis*, *matR* is a continuous reading frame within the terminal *nad1* intron *nad1i782g2* and requires RNA editing for expression. The terminal *nad1* introns in all *Salviniales* species surveyed bear some sequence similarity to *H. ensiformis matR*, but also contain ∼1.6 to 2.5 kb deletions and are largely free of RNA editing events which would generate a translatable open reading frame. This suggests that *matR* has been lost in the *Salviniales*. The details of the RNA editing analyses are presented below. While assessing mitochondrial RNA editing events in *A. filiculoides*, we noticed an unannotated transcript with low coverage (Fig. 2a), but a high density of RNA editing events (Fig. 2b). This transcript encodes two novel open reading frames resembling distinct intron maturase/reverse transcriptases (one open reading frame is encoded within an intron) (Fig. 2c). This gene is present (but previously unrecognized) in all the *Salviniales* ferns and also in *H. ensiformis* and *Dryopteris crassirhizoma*, but is absent from the *Ophioglossum californicum* and *Psilotum nudum* mitochondrial genomes (Guo et al. 2017). We searched but were unable to find orthologs in any other eukaryotic lineages. Due to the apparent confinement of this gene to leptosporangiate ferns, we have named it *mat-Lepto*. The complement of *Salviniales* mitochondrial genes are summarized and compared to *P. nudum*, *O. californicum*, and *H. ensiformis* in Table S1.

### RNA Editing in *Salviniales* Chloroplast Genomes and Mitochondrial Protein-Coding Genes

**Fig. 3.**
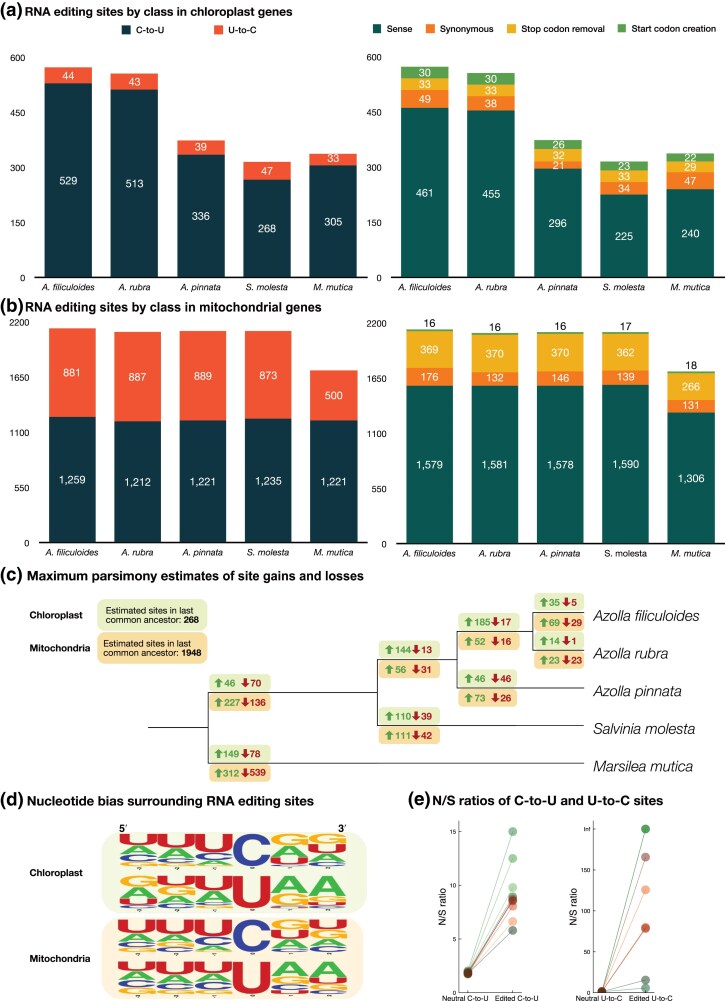
RNA editing in *Salviniales* chloroplast and mitochondria transcripts. a) Number of chloroplast C-to-U and U-to-C RNA editing sites in protein-coding genes (left) and proportion of sense, synonymous, and translation-enabling editing events in protein-coding genes (right). Sense edits are the most abundant type of RNA editing event in *Salviniales* chloroplast transcripts, but editing site abundance varies between the five species. In contrast, the number of translation-enabling editing events is much more similar between the five species. b) Number of C-to-U and U-to-C mitochondrial RNA editing events in protein-coding genes (left) and proportions of sense, synonymous, and translation-enabling editing events (right). There are many more editing events in *Salviniales* mitochondrial transcripts than there are in chloroplast transcripts. Sense edits are the most common form of editing event, but there are substantially more U-to-C stop-codon removal translation-enabling editing events than start codon creation and stop codon creation editing events. c) Maximum parsimony estimates of editing site gains and losses in chloroplast and mitochondrial transcripts. Where possible solutions were equally parsimonious, events were randomly distributed according to branch length. Maximum parsimony estimates of RNA editing gains and losses by site class are provided in Table S3. d) Frequency plots of nucleotides at positions −3 and +2 relative to C-to-U and U-to-C RNA editing sites in chloroplasts and mitochondria. Flanking nucleotide identities show a bias of U>C>A>>G in the −1 position relative to the C-to-U editing sites, which is typical of organelle RNA editing sites in other lineages. U-to-C surrounding nucleotide bias differs slightly, displaying a preference for U>A>C>>G in the −1 position relative to the edit site. Sequence logos were generated using WebLogo (Crooks et al. 2004). e) Ratios of the numbers of nonsynonymous and synonymous editing sites in water fern organelles. “Neutral” ratios are those predicted if all C or U sites in mRNA coding sequences are editable. The “edited” ratios are those observed in RNA-seq data. Green points indicate chloroplast ratios, and orange points indicate mitochondrial ratios. Color shades indicate species (from light to dark: *A. filiculoides*, *A. rubra*, *A. pinnata*, *S. molesta*, *M. mutica*). In all cases, the observed ratios are higher than the expected “neutral” ratios. The data are in Table S4.

*Salviniales* ferns contain extreme levels of organelle RNA editing. Previous estimates of 1710 RNA editing sites in the *A. filiculoides* organelle genomes were based on incomplete mitochondrial gene complements (Li et al. 2018). Reanalysis of *A. filiculoides* organelle RNA editing shows at least 3405 RNA editing sites and probably more than this due to missing intergenic mitochondrial DNA sequences. Numbers of RNA editing events, while present at extreme levels in all five *Salviniales* species, show variation between species and also between chloroplasts and mitochondria. Organelle RNA editing events are presented in Fig. 3a (plastid) and Fig. 3b (mitochondria) and summarized in Tables S2 and S3. Maximum parsimony estimates of RNA editing site gains and losses are shown in Fig. 3c. The *Azolla* chloroplasts have overall higher levels of RNA editing than *Salvinia* and *Marsilea*; however, there is also variation in the number of editing sites between *A. filiculoides*, *A. rubra*, and *A. pinnata*. RNA editing is much more abundant in mitochondrial protein-coding transcripts than plastid protein-coding transcripts for all of the *Salviniales*. Mitochondrial editing sites levels are similar between *Azolla* species and *S. molesta*, with ∼400 fewer sites present in the mitochondrial protein-coding transcripts of *M. mutica*. Although the total number of editing sites varies, the proportions of different types of editing events are more stable across all five species.

Editing sites in chloroplast and mitochondria typically show bias for which position of a codon is edited (Gray and Covello 1993; Giegé and Brennicke 1999). In all five *Salviniales* species, C-to-U sites are commonly in the second position of codons. U-to-C sites are usually at the first position in codons because the majority of U-to-C editing events remove premature stop codons (i.e. edit UAA, UAG, or UGA codons). More than two-thirds of the U-to-C events at position 1 of codons are stop codon removal events. C-to-U sites show typical preferences for the base preceding an edit site being U>C>A>>G. U-to-C sites show a similar bias for U at the preceding base; however, A is preferred to C: U>A>C>>G (Fig. 3d).

Editing events are typically classified as *synonymous* when a C-to-U or U-to-C edit in a coding sequence has no effect on the translation of the codon, *nonsynonymous* when the edit does affect the translation of the codon, and (identified but excluded from analysis) *intergenic or intronic* C-to-U or U-to-C editing events outside of protein-coding sequences. The ratio of nonsynonymous to synonymous edits (N/S ratio) is compared to expectations in Fig. 3e, where the neutral expectations assume that all C or U nucleotides in coding sequences are editable. In both organelles and in all species, N/S ratios are strongly biased toward nonsynonymous edits, particularly for U to C events.

We classified nonsynonymous RNA editing events into categories according to the effect the editing event has on the translated peptide. These categories are: (i) *start codon creation*, via C-to-U editing of ACG→AUG codons, or, in rare cases, GCG→GUG; (ii) *stop codon removal* where U-to-C editing removes premature stop codons from coding sequences; and (iii) *sense* edits where either C-to-U or U-to-C RNA editing alter a codon to encode a different amino acid (or a stop codon). Start codon creation and stop codon removal editing events have a direct effect on the capacity for a transcript to be translated. We refer to these two categories of editing events as “translation-enabling” sites.

**Fig. 4.**
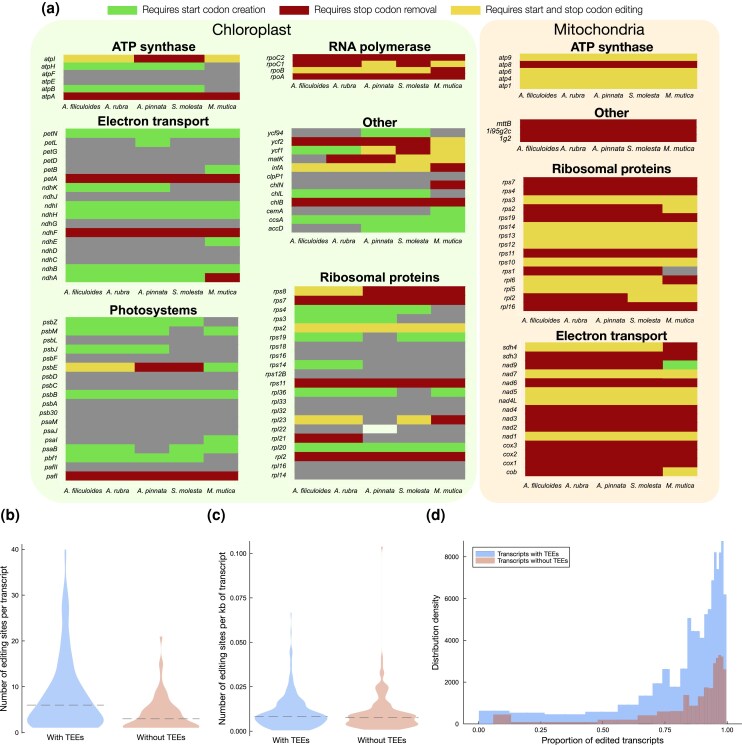
Translation-enabling editing events in chloroplast and mitochondrial protein-coding genes. a) More than half of all genes in chloroplasts, and all genes in mitochondria except *M. mutica rps1*, require start codon creation (green), stop codon removal (red), or both (yellow). b) Chloroplast genes encoding translation-enabling edits (TEE) events contain more editing sites than genes that do not. Dashed line indicates the median. c) Number of editing sites per kilobase of transcript in chloroplast genes with (blue) and without (orange) TEEs. Dashed line indicates the median. d) Chloroplast transcripts with (blue) and without (orange) TEEs are both highly edited.

In the *Salviniales* chloroplasts, more than half of protein-coding genes require start codon creation, or stop codon removal, or both for translation (Table S2). In the mitochondria, every gene except for *rps1* in *M. mutica* requires either start codon creation or stop codon removal (Fig. 4a). Translation-enabling sites in mitochondrial and chloroplast protein-coding genes are remarkably stable. In mitochondria, approximately 70% of start codon creation (*n* = 20) and stop codon creation events (*n* = 17) are conserved in all five species. Of the mitochondrial stop codon removal events, 58.25% (*n* = 400) are conserved in all five species. Comparatively, sense editing events in mitochondrial protein-coding sequences are ∼42% conserved (*n* = 2,044), and just ∼5.8% of synonymous events are shared across species (*n* = 362). In chloroplasts, 44% of start codon creation events (*n* = 18) and 40% of stop codon removal events (*n* = 45) are conserved in five species. Chloroplast genes with translation-enabling RNA editing events contain more editing sites overall than those that do not, though the proportion of editing is consistently high in genes with and without translation-enabling RNA editing sites (Fig. 4b).

### Comparative Analysis of RNA Editing Sites Shows Possible Selection for Sites That Influence Translation

**Fig. 5.**
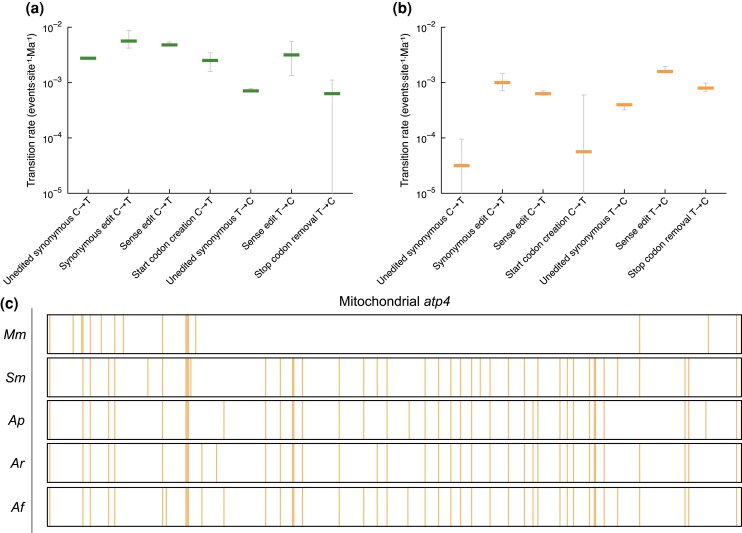
Pyrimidine transition rate analyses and clustered losses of RNA editing in *Salviniales* chloroplast and mitochondrial genes. a) Profile likelihoods of RNA editing site classes in chloroplasts shows different rates of pyrimidine transition for different classes. 95% confidence intervals show lower mutation rates for start codon creation and stop codon removal events showing that these sites are more conserved than other nonsynonymous editing events. b) Confidence intervals for editing site classes in mitochondrial transcripts show similar trends of greater conservation of translation-enabling editing events, with a much more pronounced difference in mutation rate for stop codon removal events. c) Evidence of clustered loss of RNA editing sites in the *M. mutica* mitochondrial *atp4* gene. Orange lines indicate C-to-U or U-to-C editing sites. A cluster of presumably lost editing events in *M. mutica* supports the hypothesis that editing sites can be lost en masse by reverse transcription and subsequent recombination of edited transcripts into the organelle genome.

We took advantage of having data for five fern species with well-established dated phylogenetic relationships (Fig. 1) to look at how the patterns of editing sites have evolved in this lineage. We were particularly interested in whether the rate of loss of translation-enabling sites differed from the rate of loss of other editing sites. First, sites were filtered to only retain those where editing differences between species were due to DNA mutations at the editing site (rather than differences in the specificity of the editing machinery). The sites were further filtered to only retain those where the corresponding DNA base in all species was either C or T, such that only pyrimidine transitions needed to be considered. This left 970 (900 C-to-U; 70 U-to-C) sites for analysis in the chloroplast data and 2816 (1837 C-to-U; 979 U-to-C) sites in the mitochondrial data (Table S5). Assuming the phylogeny and dating of divergences in Fig. 1 and that mutation rates have been constant throughout this time in all branches, the likelihood of each possible evolutionary trajectory leading to the observed presence/absence pattern of editing at a specific site can be estimated using just three parameters: the probability that the site in the common ancestor was edited and the gain and loss rates for editing sites. Given the filtering of the sites described above, this equates to pyrimidine transition rates at the DNA level. Log-likelihood differences favored models in which events were grouped into classes based on their translational impact, implying significant differences in the transition rates between these classes (Table S6). Confidence intervals for each transition rate (Fig. 5a and b) were calculated from profile likelihoods (Figure S7). The analysis shows that transition rates corresponding to loss of editing sites at translation-enabling sites were substantially (and often significantly, in a statistical sense) lower than loss rates at sense sites. Loss rates at sense sites were in turn lower than those at synonymous sites. Perhaps surprisingly, pyrimidine transitions at unedited sites occurred at significantly lower rates than those at edited sites, particularly in mitochondrial sequences (Fig. 5b). This implies either selection against editing, thus favoring fixation of mutations, or an acceleration of the mutation rate at edited sites. One route by which the latter might occur is via recombination with reverse-transcribed edited cDNA, which would be predicted to result in clustered losses of editing sites. Evidence in favor of this is seen in comparisons of the numbers of editing sites per coding sequence between species (Table S7), particularly between *M. mutica* and the other four ferns. For example, clustered losses of editing sites are seen in the *Marsilea* mitochondrial *atp4* gene (Fig. 5c) and other transcripts (Table S7). However, evidence for clustered loss of RNA editing in chloroplasts is less apparent between the five species examined.

Alternative explanations for the apparent conservation of translation-enabling sites were also considered. The hypothesis that edited sites revert at higher frequency because of reverse-transcription-mediated mutation raised the possibility that reversion rates could be position-dependent. Start codon creation events obviously have a strongly biased distribution (Figure S7) but partitioning sense editing events into those close to the start codon (within the first 13 codons) and those further away showed no significant difference in reversion rates (Table S6). Stop codon removal events do not show a strongly biased distribution (Figure S7), so slow reversion rates cannot be due to their position within the coding sequence. The codon sequence itself may bias reversion rates, so to test this, unedited sites were partitioned into those in an ACG context or not (no significant difference was found in either organelle, Table S5), or a TAA/TGA/TAG context or not. In the latter case, a significant difference in mutation rates was found in chloroplasts (Table S6), but Ts in a TAA/TGA/TAG context mutated to C at a higher rate than those in other contexts, i.e. the opposite effect to that observed at stop codon removal sites.

### RNA Editing as a Lever of Control for Translation

**Fig. 6.**
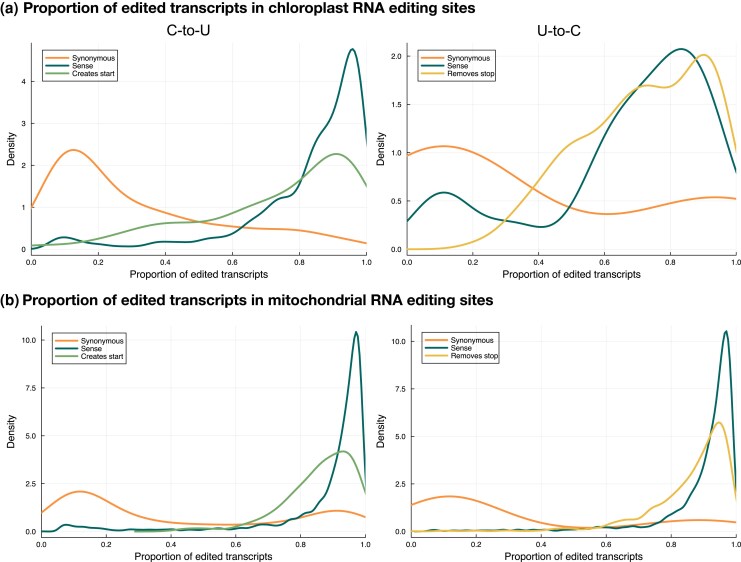
Different classes of RNA editing events are edited at different proportions. a) Editing patterns for sites in chloroplasts. The plots show kernel density estimates for the distributions of the proportion of transcripts that are edited at each site. Sense and translation-enabling editing sites are generally very highly edited whereas synonymous sites are generally edited at low proportions. Start codon creation events in chloroplasts are generally edited in lower proportions than sense sites (*P*-value 2.6e-7, one-tailed Mann–Whitney *U* test). b) Editing patterns for sites in mitochondria show similar patterns for sense, translation-enabling, and synonymous editing sites. Both start codon creation and stop codon removal events in mitochondria are generally edited in lower proportions than sense sites (*P*-values 7.7e-7 and 2.5e-54, respectively, one-tailed Mann–Whitney *U* test).

The higher degree of conservation of translation-enabling RNA editing sites suggests that these sites may be playing a role in controlling translation. Plotting the proportion of edited transcripts for translation-enabling editing sites alongside synonymous and sense events shows the majority of nonsynonymous editing events are converted at an average of ∼95% in both chloroplasts (Fig. 6a) and mitochondria (Fig. 6b). Synonymous editing events are edited at a much lower average proportion (Fig. 6a). This could indicate that many synonymous editing events are low efficiency off-target RNA editing events of proteins making other types of editing events at alternate sites. Stop codon creation events in both chloroplast and mitochondria show similar conversion proportions as sense editing events, but both start codon creation events and stop codon removal events have broader frequency distributions (Fig. 6a and b). In both chloroplasts and mitochondria, the proportion of edited transcripts for start codon creation events shows two clear peaks at ∼0.5 and ∼0.9. Examination of Illumina reads including a start codon editing site and downstream editing sites within the same read indicate that start codon editing events may occur late in transcript processing (Table S8).

### Shotgun Proteomics of *M. mutica* Thylakoids

To verify some of our assumptions regarding the translational significance of the observed editing events, we carried out a proteomics analysis of thylakoid-enriched proteins extracted from *M. mutica* chloroplasts. Thylakoids were chosen as a readily available source of abundant organelle-encoded proteins. Tryptic digests were analyzed by mass spectrometry. Peptide masses were matched against protein sequences translated from unedited or edited *Marsilea* chloroplast ORF sequences. In all, 448 peptides corresponding to *Marsilea* chloroplast proteins were identified using a q-value threshold of 0.01. As would be expected, coverage was greater (∼50%) for abundant relatively soluble proteins such as RbcL and ATPase F1 subunits but much lower for rarer hydrophobic proteins such as NdhB or NdhD. Seventeen nonsynonymous editing events are within the coding regions covered by these peptides (Table 2). This peptide data supports several long-held assumptions lacking previous experimental evidence. The peptide MFSTLTAFR from NdhI confirms that the ACG start codon is translated as methionine, presumably following editing to AUG. Three peptides confirm that U to C editing of premature stop codons allows them to be read through with insertion of glutamine. This confirms that the edited base in U-to-C editing is read as a C by the chloroplast translation machinery. In general (16/17 cases), only the peptide corresponding to the edited mRNA is detected. The exception is the atpEeU308SL event (edited at ∼40%) where both the serine-containing and leucine-containing peptides are detected with high confidence.

**Table 2:** Editing sites captured in *M. mutica* thylakoid shotgun proteomic**s**

Gene	Site ID	Proportion edited	Codon	Edited codon	AA	Edited AA	Edited peptides	Posterior error probability (edited)	Unedited peptides	Posterior error probability (unedited)
*atpA*	atpAeC103*Q	0.97	UAA	CAA	*	Q	VVNIGTVL**Q**VGDGIAR	1.76e-05		
*atpB*	atpBeU503SL	1.00	UCA	UUA	S	L	IG**L**FGGAGVGK	5.41e-04		
*atpB*	atpBeU590SF	1.00	UCC	UUU	S	F	AHGGVSV**F**GGVGER	5.96e-06		
*atpE*	atpEeU308SL	0.40	UCA	UUA	S	L	KAFQAAQAD**L**AK, AFQAAQAD**L**AK	2.40e-08	AFQAAQAD**S**AK, KAFQAAQAD**S**AK	2.64e-05
*atpI*	atpIeU481HY	0.99	CAU	UAU	H	Y	GLS**Y**FGK	1.10e-01		
*ndhB*	ndhBeU779PL	0.97	CCG	CUG	P	L	VAA**L**ALSTR	6.87e-03		
*ndhF*	ndhFeU1153LF	0.95	CUC	UUC	L	F	SQNML**F**MGGLR, SQNM*L**F**MGGLR, SQNML**F**M*GGLR, SQNM*L**F**M*GGLR	1.34e-10		
*ndhF*	ndhFeU1505TI	0.15	ACA	AUA	T	I	EA**I**DPPKESLIHNETDKTDSNVSPK	1.00e-01		
*ndhH*	ndhHeU317PL	0.84	CCU	CUU	P	L	VIM**L**ELSR, VIM*LELSR	1.51e-02		
*ndhI*	ndhIeU2TM	0.48	ACG	AUG	T	M	**M** FSTLTAFR, M*FSTLTAFR	1.39e-03		
*ndhI*	ndhIeU14PL	0.84	CCA	CUA	P	L	**M** FSTLTAFR, M*FSTLTAFR	1.39e-03		
*ndhI*	ndhIeU404SL	0.99	UCG	UUG	S	L	HE**L**NYDQTALGR	2.02e-04		
*pafI*	pafIeC166*Q	0.95	UAA	CAA	*	Q	DGMSAQSEGEYAEAL**Q**NYYK	8.59e-03		
*petA*	petAeC271*Q	0.92	UAG	CAG	*	Q	IPYDT**Q**IK	7.00e-02		
*rpoC2*	rpoC2eU3512TI	0.95	ACU	AUU	T	I	SGD**I**IQGLPK	3.68e-01		
*rps7*	rps7eU365SL	0.99	UCG	UUG	S	L	**L** SDELMDAAR	8.41e-02		
*ycf1*	ycf1eU676LF	0.72	CUU	UUU	L	F	APVP**F**LTR	1.59e-01		

Nonsynonymous editing sites in peptides of *M. mutica* thylakoid-enriched proteins include stop codon removal events and start codon creation events. Peptides are only present in their edited state in all proteins except for products of *atpE* which are also present in an unedited state. Asterisks (*) in the AA column indicate stop codons. Bold underlined amino acids in edited peptide sequences are translated from edited codons. M* indicates oxidised methionine.

### Identification of RNA Editing Proteins in *Salviniales* Transcriptomes

Transcriptomes assembled for *A. rubra*, *A. pinnata*, *S. molesta*, and *M. mutica* were assessed for completeness by a benchmarking universal single-copy orthologs (BUSCO) analysis referencing the embryophyta_odb10 database (Simão et al. 2015; Manni et al. 2021; Kuznetsov et al. 2023). Analysis of protein sequences translated from open reading frames indicate transcriptome completeness between 80% and 88%, though overlap of missing BUSCOs may be uniformly absent in the fern genomes, and therefore, completeness may be higher than estimated (Figure S8). The high completeness of the transcriptome assemblies makes them suitable for screening for putative PPR editing factors.

**Fig. 7.**
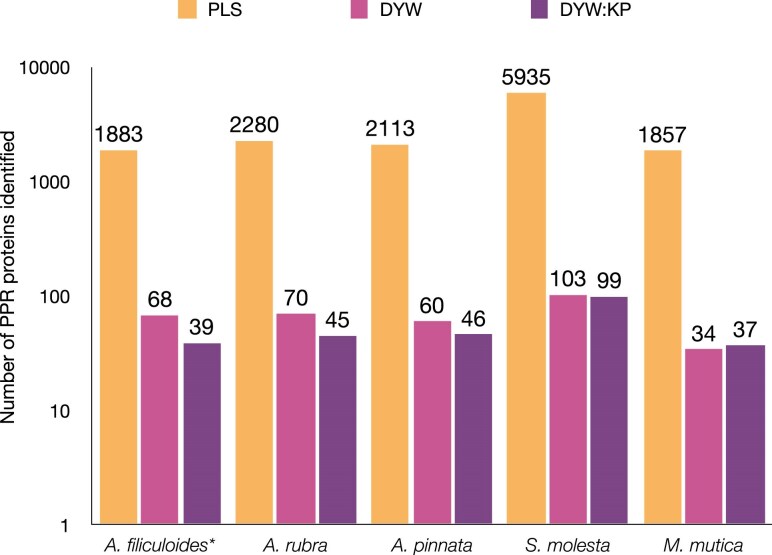
PPR protein sequences identified in the transcriptomes of *A. rubra*, *A. pinnata*, *S. molesta*, and *M. mutica* and in the nuclear genome assembly of *A. filiculoides*. There are many more PLS protein sequences than sequences containing C-to-U or U-to-C editing domains. Asterisk (*) indicates protein sequences that were identified in a nuclear genome.

The genome of *A. filiculoides* and de novo transcriptome assemblies for *A. rubra*, *A. pinnata*, *S. molesta*, and *M. mutica* were scanned for PPR protein sequences using hidden Markov models for DYW and DYW:KP PPR editing factors, described in Gutmann et al. (2020). We identified many thousands of putative PPR proteins. The greater number of protein sequences identified in *S. molesta* is likely due to its allopentaploid nuclear genome (Sigel et al. 2025). We compared the number of *S. molesta* proteins to protein sequences identified in the genome of *S. cucullata* (Li et al. 2018), which encodes for similar numbers of proteins to the other *Salviniales* species (Figure S9). However, only a small proportion of predicted proteins contain the C-terminal DYW or DYW:KP domains associated with C-to-U and U-to-C editing, respectively (Fig. 7). This small number of “full-length” editing proteins cannot account for the many thousands of RNA editing events we see in the mitochondria and chloroplasts. In *Arabidopsis thaliana*, short PLS proteins with DYW domains, or DYW domains with no PPR motifs at all, act in trans with PLS PPR proteins to facilitate C-to-U RNA editing at multiple sites in both the chloroplast and mitochondria (Boussardon et al. 2012; Andrés-Colás et al. 2017; Diaz et al. 2017). The abundance of PLS protein sequences in *Salviniales*, and the presence of short DYW and DYW:KP protein sequences, is highly suggestive of both C-to-U and U-to-C editing being facilitated by domains acting in trans with PLS PPR proteins. Prediction of which PLS protein interacts with a putative DYW or DYW:KP donor domain is challenging with our current understanding of these types of protein–protein interactions and no obvious protein sequence signatures at the C-terminal ends of PLS PPR protein sequences.

## Discussion

### A Novel Gene in Leptosporangiate Fern Mitochondria

The *mat-Lepto* gene encoded in leptosporangiate fern mitochondria has not been previously described. There are very few complete fern mitochondrial genomes available but complete assemblies have been constructed for eusporangiate ferns belonging to the *Ophioglossidae* (Guo et al. 2017; Hao et al. 2022) and the leptosporangiate ferns *D. crassirhizoma* (*Song* et al. *2021*) and *H. ensiformis* (Zumkeller et al. 2023). Our investigation of these mitochondrial genomes showed an absence of *mat-Lepto* in eusporangiate mitochondrial genomes and its presence in leptosporangiate mitochondrial genomes. The low expression level of *mat-Lepto* (Fig. 2a) may explain why it has been missed previously. Although this low RNA-seq read depth complicates the detection of RNA editing events, all 42 premature stop codons could be shown to be removed to allow for the translation of Mat-Leptoi95g2c and Mat-Lepto (Fig. 2c).

The origins of *mat-Lepto* are unclear. Downstream of *mat-Lepto* is a partial repeat of *rps14* exon 2. This arrangement implies that *mat-Lepto* was once nested within the intron of *rps14*, making it formerly a “twintron” (Zumkeller et al. 2020). The *mat-Leptoi95g2* intron contains a region with high sequence similarity to part of *nad5* intron 4, suggesting that the two introns share a recent common ancestor. In our searches through the limited mitochondrial genomes available, Mat-Leptoi95g2c and MatLepto are always present together. Comparison of *mat-Lepto* to a database of mitochondrial group II intron families showed no orthologous sequences in the eukaryotic lineage outside of the leptosporangiate ferns (Zumkeller and Knoop 2023). Mat-Leptoi95g2c and Mat-Lepto both show conserved RT domains (Blocker et al. 2005), and Mat-Lepto also encodes a C-terminal X-domain associated with endonuclease activity (San Filippo and Lambowitz 2002). Both maturases appear to contain the necessary features for splicing. Presumably, Mat-Leptoi95g2c is required for splicing of its host intron (and possibly *nad5* intron 4, given the homology between the two introns), while Mat-Lepto is likely to be required for splicing of other mitochondrial introns, of which the *rps14* intron would be the most obvious candidate. Further characterization would be required to determine the precise activity of these maturases.

### Idiosyncratic Evolution of RNA Editing in Hyper-editing Species

*Salviniales* ferns comfortably merit the label of “hyper-editors,” with more than 3400 RNA editing sites in the organelles of some species. The *Salviniales* contain the most described RNA editing sites of any eusporangiate or leptosporangiate fern studied to date (Guo et al. 2015, 2017; Knie et al. 2016; Li et al. 2018; Zumkeller et al. 2023; Peng et al. 2024), albeit with the distinct advantage of having assembled mitochondrial genes to assess. In *Salviniales*, C-to-U editing exceeds U-to-C editing, and editing in the mitochondria exceeds that in the chloroplast. We have seen evidence that the loss of RNA editing can happen in clusters (Fig. 4c), possibly as a result of recombination of edited cDNA into the organelle genomes, but this alone does not explain the large variation in RNA editing between species. Lineages of nonseed plants seem to be on individual evolutionary trajectories of gaining or losing C-to-U and U-to-C editing sites and often at different rates in chloroplasts and mitochondria. For example, we can compare the *Salviniales* to other hyper-editing lineages such as the hornwort *A. agrestis* (Gerke et al. 2020) and the lycophytes *Selaginella moellendorffii* (Hecht et al. 2011) and *S. uncinata* (Oldenkott et al. 2014). RNA editing in *A. agrestis* has evolved along two opposite trajectories relative to the *Salviniales.* In *A. agrestis*, U-to-C editing exceeds C-to-U editing, and editing in chloroplasts exceeds that in the mitochondria (Gerke et al. 2020). Levels of RNA editing in *Selaginella* vary greatly among species (Smith 2020). *Selaginella uncinata* contains more than 3400 sites in its chloroplast transcriptome alone (Oldenkott et al. 2014), whereas *Selaginella lepidophylla* contains a modest 720 sites (Smith 2020). U-to-C editing is absent entirely in *S. moellendorffii* (*Hecht* et al. *2011*) and most likely in all other *Selaginella* species (Oldenkott et al. 2014; Smith 2020), but is present in other lycophytes such as *Isoetes engelmannii* (Grewe et al. 2011) and in *Huperzioid* lycophytes (Kwok van der Giezen et al. 2024). Another divergent feature of *A. agrestis* is the prevalence of PPR proteins encoding DYW:KP C-terminal domains. Rather than a small number of DYW:KP domains that potentially act in trans, like we see in the *Salviniales*, there is a strong correlation of the number of full-length DYW:KP proteins and U-to-C editing sites in *A. agrestis* (Gutmann et al. 2020). However, like we see in *Salviniales*, there are only a handful of DYW domains in *A. agrestis*, which may act in trans to edit C-to-U. Unsurprisingly, *Selaginella* species do not encode any DYW:KP proteins for U-to-C editing (Gutmann et al. 2020). The evolutionary trajectory of RNA editing in plants may to a large extent reflect the evolutionary trajectory of the genes encoding RNA editing PPR proteins in the nuclear genome (Wu and Chaw 2022).

### Alternative Models for Adaptive and Nonadaptive RNA Editing

The most widely accepted explanation for the prevalence of RNA editing is CNE, where RNA editing is a neutral process resulting from mutations in genes for existing RNA editing machinery “presuppressing” deleterious mutations in organelle genes before they arise (Gray 2012). However, numerous proposals have been made that, under some circumstances, RNA editing may provide selective advantages, either for the organelle in which it occurs, or for the entire cell/organism. For example, it has been suggested that RNA editing may play a role in nucleocytoplasmic conflicts where organellar mutations increase organellar reproductive fitness at the expense of nuclear reproductive fitness (Castandet and Araya 2012); in such cases, RNA editing may be selected for if nucleus-encoded factors can suppress the organellar mutations. Such conflicts are the basis for CMS and fertility restoration in flowering plants (Budar et al. 2003). However, in most cases, CMS-inducing mutations are not point mutations and fertility restoration factors induce RNA cleavage, not RNA editing (Melonek et al. 2021). Rare exceptions where RNA editing may be implicated have been reported (Howad and Kempken 1997). In another case of potential selective advantage, RNA editing has been proposed as a mechanism to posttranscriptionally activate gene expression through creation of start codons or removal of stop codons, as we have investigated in the *Salviniaceae*. A compelling example has been reported in *Fusarium*, where stop codon removal by A-to-I editing promotes expression of mRNAs implicated in sexual development (Qi et al. 2024). The aforementioned cases are examples of restorative editing where the editing tends to restore the ancestral sequence. The high specificity of plant organellar editing enzymes may drive the evolution of restorative RNA editing in these organelles (Sloan 2025). Experiments expressing PPR RNA editing factors in plant and human cytosol resulted in high numbers of off-target editing events (Lesch et al. 2022; Thielen et al. 2024) suggesting evolution of restorative editing in these compartments may be unlikely because of unavoidable deleterious off-target editing (Sloan 2025). Instead, nuclear and cytosolic A-to-I editing tends to act to diversify transcriptome sequences (reviewed in Eisenberg and Levanon (2018) and Xin et al. (2025)). Although as in restorative editing, the vast majority of editing events are likely to be neutral (at best), some events are potentially advantageous in certain situations. Examples include the Q586R site of the GluA2 subunit of AMPA glutamate receptors in vertebrates (Wright et al. 2023) (which may be the only essential nonsynonymous A-to-I edit in mammals (Chalk et al. 2019)) and abundant conserved nonsynonymous A-to-I editing sites in coleoid cephalopods (Jiang and Zhang 2019).

Whether or not RNA editing is adaptive in A-to-I editing systems has been studied by looking for positive selection of nonsynonymous events compared to synonymous events (e.g. Qi et al. (2024)). A signature of adaptive editing is a high N/S ratio compared to expectations assuming neutral selection (Duan et al. 2017). The N/S ratios at editing sites in plant organelles tend to be extremely high (see e.g. Fig. 3e), but alternative explanations besides an adaptive advantage to nonsynonymous editing are possible. There is a major mechanistic difference between plant organellar RNA editing and the A-to-I editing seen in animals and fungi. In A-to-I editing, one or a few enzymes edit many sites, and evolutionary differences in editing patterns can be safely assumed to be due to mutations at the sites themselves and thus comparable between synonymous and nonsynonymous sites. In plant organelles, many enzymes each edit only one or a few sites, and many evolutionary differences in editing patterns are due to mutations in the enzyme sequences, not the target sites. For the *Salviniales* ferns described here, the total sequence length of the genes encoding the putative organellar RNA editing factors (>1 Mb) exceeds the combined length of both organellar genomes (<<1 Mb). For synonymous editing sites, reversions at the target site or mutations in the editing factors that target them can be expected to be equally selectively neutral. However, for nonsynonymous editing sites, reversions at the target site are selectively neutral, but mutations in an editing factor gene that prevent editing at the site are not, and can be expected to be strongly selected against. Thus, we can expect synonymous editing sites in plant organelles to be lost much more rapidly than nonsynonymous sites, and this expectation is supported by the much lower prevalence of synonymous sites in plant organelles (see Fig. 3e and many previous examples, e.g. in Jobson and Qiu (2008)). To attempt to minimize the complications of the differing effects of mutations within the genes encoding editing factors, we filtered all editing sites used in the evolutionary analyses to only retain those where editing status differences were explainable solely by DNA mutations at the sites themselves.

### Emergent Adaptive Roles for RNA Editing Sites Arising From CNE

We have focused on pyrimidine transition rates in the organelle genomes, which are a combined outcome of the rate of mutation and the rate of fixation of these mutations, the latter reflecting natural selection among competing variants in the population. As we are most interested in potential selection pressures and have no direct way of measuring mutation rates over evolutionary time, we have attempted to classify sites such that we can compare classes likely to have comparable mutation rates. For example, as is clear in Fig. 5a and b, transition rates are quite different between mitochondria and chloroplasts and between edited and unedited sites. We suspect that in both cases, these differences are primarily due to differences in mutation rates, not selection pressure, for example, via the propensity for edited sites to direct mutation of the genome, as illustrated in Fig. 5c, and as observed in many other plant organelles (Sloan et al. 2010). Thus, we cannot safely mix chloroplast and mitochondrial data, and we cannot safely take the transition rate at unedited synonymous sites as a “neutral” reference for those at edited sites. The next best choice as a neutral reference would be transition rates at edited synonymous sites, but there are very few such sites where the variation between species is due to DNA mutation—in nearly all cases, the DNA base at such sites is not altered, and the difference in editing must be due to a difference in the specificity of the editing machinery, consistent with the view that such sites are “off-target” events (Fig. 6) of editing factors with a primary target elsewhere. Thus, the comparison we have focused on is between translation-enabling editing events and sense editing events that alter codons without directly affecting whether or not the mRNA is translatable. With a much smaller dataset, Li et al. (2018) showed that start codon creation and stop codon removal events were more conserved between *A. filiculoides* and *S. cucullata* than other editing events and proposed that “creating the translatable sequence by RNA editing has an advantage over having it encoded by the genome,” but could not rigorously test this hypothesis with the data they had. The potentially adaptive role of translation-enabling editing events checkpointing translation has also been observed in *Pteridaceae* ferns (Fauskee et al. 2025). With our expanded dataset, we provide much stronger evidence that not only are such events more conserved than other nonsynonymous events, but that this is in large part due to a reduced rate of fixation of DNA mutations that would revert the sequence such that it no longer requires editing. We tested, and eliminated, two alternative hypotheses for the low rates of fixation of DNA mutations at these sites that posit differences in mutation rates. Firstly, although start codon creation events are strongly biased toward the 5′ end of the transcript, this positional bias cannot explain the slow reversion rate as other similarly placed nonsynonymous sites do not show such a slow rate (Figure S7 and Table S6). Secondly, nucleotide mutation rates can depend strongly on the immediate nucleotide sequence context (Waneka et al. 2025), but we found no evidence that C→T transitions in ACG triplets (at unedited sites) occur less frequently than at other sites (Table S6) and T→C transitions in TAA/TGA/TAG codons (at unedited sites in chloroplasts) occur more frequently than at other sites, not less frequently (Table S6).

In conclusion, as the tendency is clear for both mitochondria and chloroplasts, and for both start codon creation and stop codon removal (and thus for both C→T and T→C transitions) (Fig. 5a and b), we believe that the observed differences in reversion rates most probably reflect stronger selection pressure on retention of start and stop codon editing events. Why might editing start and stop codons be more advantageous for these plants than having the “correct” sequence in the genome? We speculate that one reason may be that it helps prevent premature translation of partially edited transcripts. If the start codon creation or stop codon removal tends to occur late in the processing of the mRNA (and there is evidence for this in the lower proportion of transcripts within a highly edited state at these sites, Fig. 6), then this will act to reduce premature translation. Another piece of evidence that supports this hypothesis is that highly edited transcripts are more likely to require start codon editing (Fig. 4b). A potentially more exciting possibility is that start and stop codon editing provide a “gate” to control expression of proteins in response to developmental or environmental signals.

### RNA Editing as a Mechanism for Controlling Translation

Start codon creation and stop codon removal editing events effectively create a natural logic gate within organelle transcripts. Where a gene contains only a single start codon creation editing event, like the *psaB* gene in the *Salviniales* chloroplasts, expression of the PsaB protein is dependent on the expression of the nuclear editing factor. This effectively creates a switch to control organelle gene expression. However, the more common scenario in *Salviniales* chloroplasts and mitochondria is dozens, if not hundreds of RNA editing sites being processed prior to translation. This includes, in the case of some mitochondrial transcripts, start codon creation paired with multiple stop codon removal events. In combination, this creates a multiple input “AND” gate where the expression of many PPR proteins is required for correct translation of an organelle gene product.

For editing to exert control of gene expression, at least some sites must be edited less than maximally under some circumstances. We found many such instances, and in general, both start codon creation and stop codon removal sites are less edited than other nonsynonymous sites in both organelles (Fig. 6). For example, in the *Salviniales* chloroplast *rpoB* gene, the editing efficiency of start codon creation in *A. filiculoides*, *A. rubra*, *A. pinnata*, and *S. molesta* ranges from 38% to 65%. Although *M. mutica rpoB* does not contain a start codon editing event, a downstream stop codon removal event common to all five species is consistently edited between 32% and 50%. Low and variable editing efficiency of stop codon removal events is also seen in *rpoA*, *rpoC1*, and *rpoC2*. This is consistent with a role in regulation of expression, but far from proof of it. Analysis of editing frequencies at different developmental stages or under different growth conditions may be informative.

### Compartmentalization of Transcript Processing

Analysis of *M. mutica* thylakoid fractions captured peptides illustrating the consequences of nonsynonymous editing events, including start codon creation and stop codon removal events. The majority of peptides covering edited sites correspond only to the edited transcript, with the exception of the atpEeU308SL event where peptides corresponding to both edited and unedited transcripts were seen. This site may be an exception because it is only 40% edited. Although rapid turnover of polypeptides translated from unedited or partially edited transcripts cannot be ruled out, the data strongly suggests that transcripts are generally fully edited before translation. This is also the case for the organelle proteomes of *A. thaliana* (Rugen et al. 2024; van Wijk et al. 2024). To our knowledge, this is the first analysis of organelle protein sequences in the context of RNA editing in a plant capable of U-to-C conversion. The presence of translation-enabling editing events, particularly stop codon removal by U-to-C editing, confirms prior assumptions of how edited transcripts are interpreted by translational machinery. The mechanism of U-to-C editing is not fully understood, but there is evidence that a lysine side chain of the editing enzyme may be an amine donor, or a cross-linked intermediate in an amination or transamination reaction (Hayes et al. 2024). The translation of *pafI* and *petA* in *M. mutica* chloroplasts after */Q editing confirms that ribonucleotides modified by U-to-C editing are indeed interpreted as cytidine, and not as a ribonucleotide product of uridine.

The generally complete processing of RNA in *M. mutica* chloroplasts and in *A. thaliana* (Rugen et al. 2024; van Wijk et al. 2024) prior to translation suggests that this RNA processing takes place in a compartmentalized space from which ribosomes are excluded. The requirement for start codon creation and stop codon removal may place an additional barrier that helps prevent premature translation of partially edited mRNAs. Indeed, it is conceivable that this is the major explanation for the apparent selection for retention of these editing events.

## Materials and Methods

### DNA/RNA Extraction and Sequencing

*Azolla rubra* and *A. pinnata* were sampled from the University of Western Australia taxonomic garden, with permission, and grown in separate containers under native conditions. *Marsilea mutica* was sampled from a private pond, with permission. *Salvinia molesta* is a declared pest species in Western Australia and was sampled with permission from the University of Western Australia taxonomic garden where it is kept with regulatory approval. *Salvinia molesta* was grown in a PC2-certified plant growth facility for nucleic acid extraction, and subsequently destroyed. *Azolla rubra*, *A. pinnata*, and *S. molesta* DNA was extracted from whole plants sampled from the same clonal colony using a DNeasy kit (Qiagen, Hilden, Germany) according to the manufacturer's instructions. DNA was extracted from frond tissue sampled from a single *M. mutica* rhizome using a DNeasy kit (Qiagen, Hilden, Germany). DNA was fragmented to 550 bp with a Covaris S220 focused-ultrasonicator. DNA libraries were prepared using an Illumina TruSeq Nano DNA kit (Illumina Inc., San Diego, CA, USA). DNA was sequenced by Novogene (Novogene, Singapore) on an Illumina HiSeq 2500 and yielded 20 M reads per library.

RNA from *A. rubra*, *A. pinnata*, and *M. mutica* was extracted using an RNeasy Plant Mini kit (Qiagen, Hilden, Germany) according to the manufacturer's instructions. The fibrous *S. molesta* tissue required a buffer containing guanidium hydrochloride (Qiagen RNeasy Plant Mini kit buffer RLC) for successful RNA extraction. A mix of whole plants from clonal colonies was used for *A. rubra*, *A. pinnata*, and *S. molesta*. Fronds from a single rhizome were used for *M. mutica*. RNA was depleted of ribosomal RNA using an Illumina Ribo-Zero Plus rRNA Depletion kit (Illumina Inc., San Diego, CA, USA). 150 nt paired-end RNA libraries were prepared using an Illumina TruSeq Stranded mRNA kit (Illumina, Inc., San Diego, CA, USA). RNA libraries were sequenced by Novogene (Novogene, Singapore) on an Illumina HiSeq 2500 and yielded 110 M reads per library.

### Chloroplast Genome Assembly and Annotation

DNA and RNA reads were trimmed using BBDuk from the BBtools suite (settings ktrim = r, k = 23, mink = 11, hdist = 1, ftm = 5, tpe, tbo) (Bushnell 2017). Trimmed reads for each species were used to assemble chloroplast genomes using NOVOPlasty (settings type = chloro, genome range = 120,000 to 220,000 bp, kme = 51) (Dierckxsens et al. 2016). The *A. filiculoides rbcL* gene was provided as a seed sequence for *A. rubra* and *A. pinnata* chloroplast genome assembly, and the complete *A. filiculoides* chloroplast genome (GenBank accession MF177094) was provided as a reference sequence. The *S. cucullata rbcL* gene was provided as a seed sequence for *S. molesta* chloroplast genome assembly, and the complete *S. cucullata* chloroplast genome (GenBank accession MF177095) was provided as a reference sequence. The *Marsilea crenata rbcL* gene was provided as a seed sequence for *M. mutica* chloroplast genome assembly, a reference chloroplast genome was not provided. Assembled chloroplast genomes were polished using Pilon (Walker et al. 2014), but no improvements were made. tRNAscan-SE 2.0 (Chan et al. 2021) was run on the assemblies. tRNAs identified by tRNAscan-SE 2.0 were verified by cross-referencing against the PlantRNA2.0 database (Cognat et al. 2022). Chloroplast genomes were also assembled using GetOrganelle (Jin et al. 2020), which produced identical assemblies.

The chloroplast genomes were annotated in two stages using a prototype version of Chloë (https://github.com/ian-small/chloe). The first stage was a direct annotation of the genome assemblies produced by NOVOplasty. After identification of RNA editing sites in the chloroplast genomes, “edited” copies of the chloroplast genomes were created using a custom Julia script called edit_genome.jl. The edited assemblies were reannotated with Chloë and then curated to ensure start and end annotations were correct accounting for RNA editing creating start codons and stop codons, or removing stop codons. Corrected annotations were then applied to the original chloroplast genome assembly files. The *A. filiculoides* (GenBank accession MF177094), *A. rubra*, *A. pinnata*, *S. molesta*, and *M. mutica* chloroplast genomes were aligned using MAFFT v7.450 (Katoh and Standley 2013) in Geneious Prime v.2023.0.4, and annotations were compared with a focus on start and stop coordinates. Introns and pseudogenization events were individually verified with sequence data.

### Mitochondrial Contig Assembly and Annotation

*Azolla rubra*, *A. pinnata*, *S. molesta* and *M. mutica* DNA reads were mapped to *A. filiculoides* mitochondrial contigs (GenBank accessions MN400566-MN4000574) (Feng and Wicke 2022). Mapped DNA reads were assembled with SPAdes (v3.12.0) (Bankevich et al. 2012). Assembly graphs were visualized in Bandage (Wick et al. 2015) and contigs referenced to *A. filiculoides* mitochondrial gene using the Bandage wrapper for NCBI BLAST. Contiguous sequences for individual genes were extracted and manually curated. *Azolla filiculoides* annotations were transferred to the extracted mitochondrial contig sequences using Geneious Prime (v2024.0.5). RNA editing sites were identified and “edited” copies of the contigs were generated using the Julia script edit_genome.jl. Annotations were curated to correct for start and stop coordinates accounting for RNA editing. Intron splice junctions were verified using sequence data.

### Detection of RNA Editing Events

Trimmed RNA reads for each *Salviniales* species were merged using BBmerge (settings qtrim = 2, trimq = 10,15,20, minq = 12) (Bushnell 2017). Merged and unmerged reads were mapped to the chloroplast genomes and mitochondrial contigs using BBwrap (settings mappedonly = t, ambiguous = random) (Bushnell 2017). RNA nucleotide count files at every position in the genomes (chloroplast) or contigs (mitochondria) were generated for each species using Pyrimid (settings -m 0, -u) (https://github.com/ian-small/pyrimid). RNA count files were subjected to binomial tests at each site to identify sites where editing exceeds an arbitrary threshold of 5%. *P*-values were corrected for multiple testing using the Benjamini–Hochberg procedure. A full list of RNA editing sites in the *Salviniales* species used in this study is provided in Table S5.

### Profile Likelihood Analysis of Pyrimidine Transition Rates

There are five species in the analysis, thus 2^5^ (32) possible editing patterns. There are nine branches in the phylogenetic tree (Fig. 1) including the branch leading to the last common ancestor of the *Salviniales*, so 2^9^ (512) possible evolutionary trajectories to reach these 32 potential editing patterns. The likelihood of each trajectory was calculated using estimated gain and loss rate parameters. The likelihood of each pattern was calculated by summing the likelihood of each trajectory leading to the pattern. The overall likelihood of the data was calculated as the product of the likelihoods of each observed pattern. The gain and loss rate parameters (and the probability that the ancestral site was edited) were estimated by calculating profile likelihoods over the plausible ranges for each parameter. Confidence intervals (95%) for the loss rates were estimated as the range over which the profile likelihoods exceeded the maximum likelihood −1.92. The code for this analysis is available at 10.5281/zenodo.14511549.

### Transcriptome Assembly

Using trimmed RNA reads, de novo transcriptome assemblies were produced for *A. rubra*, *A. pinnata*, *S. molesta* and *M. mutica* using rnaSPAdes (Bushmanova et al. 2019). Open reading frames were produced in all six reading frames using the Julia script orfinder.jl. The completeness of the transcriptomes was assessed by BUSCO protein analysis using the embryophyta_odb10 dataset (Simão et al. 2015; Manni et al. 2021; Kuznetsov et al. 2023).

### Identification of PPR Proteins

Open reading frames in de novo transcriptome assemblies for *A. rubra*, *A. pinnata*, *S. molesta* and *M. mutica*, and from the nuclear genome of *A. filiculoides*, were searched for PPR proteins using “hmmsearch” (HMMER v3.2.1) and the DYW/DYW:KP hidden Markov model profile described in (Gutmann et al. 2020). PPRfinder (Gutmann et al. 2020) was run on the hmmsearch output to annotate PPR protein motif structure.

### Shotgun Proteomics

Open fronds of *M. mutica* growing in a pond were collected at the end of the night. Thirty grams of fronds was washed in cold water, cut with razor blades, and ground in 180 mL of ice-cold grinding buffer (0.3 M mannitol; 50 mM HEPES-NaOH, pH 8.0; 10 mM EDTA; 8 mM 2-mercaptoethanol) with 0.1% (w/v) bovine serum albumin and 0.6% (w/v) polyvinylpyrrolidone (40,000) in a Braun 4184 blender (3 × 5 s bursts) (Hegeman et al. 2005). The slurry was filtered through four layers of cheesecloth and spun three times at 6,000 *g* for 1 s to remove the starch. The supernatant was centrifuged at 10,000 *g* for 10 min. This crude thylakoid pellet was washed with the grinding buffer (without BSA and PVP) twice and pelleted at 10,000 *g* for 10 min. The proteins were extracted with the methanol-chloroform method and digested with trypsin prior to concatenated high pH fractionation and mass spectrometry analysis according to Duncan et al. (2017) on an Agilent 6550 QTOF LCMS with C18 reversed phase chromatography by an Agilent 1200 series nano HPLC. Raw data were converted to *.mzML format using Msconvert (Kessner et al. 2008) and MS2 spectra matched against a custom peptide database derived from edited and unedited sequences using Comet (Eng et al. 2013). Results were post-processed with Percolator V3.07.1 (Käll et al. 2007). Spectra corresponding to edited sites were extracted using the mzR library (Chambers et al. 2012) (Figure S10). The mass spectrometry proteomics data have been deposited to the ProteomeXchange Consortium via the PRIDE (Perez-Riverol et al. 2025) partner repository with the dataset identifier PXD065205.

## Supplementary Material

msaf241_Supplementary_Data

## Data Availability

Raw sequence data for *A. rubra*, *A. pinnata*, *S. molesta*, and *M. mutica* is available on the NCBI Sequence Read Archive (SRA) database under BioProject accession PRJNA1185439. Chloroplast genome assemblies are available in GenBank as accessions PQ616047 to PQ616050. An updated annotation for *A. filiculoides* (MF177904) is provided as Supporting information SI1. Mitochondrial genes are available on GenBank under accessions PQ554530 to PQ554566 (*A. rubra*), PQ554567 to PQ554603 (*A. pinnata*), PQ554604 to PQ554640 (*M. mutica*), and PQ554641 to PQ554677 (*S. molesta*). Transcriptome assemblies have been deposited at DDBJ/EMBL/GenBank under the accessions GLAU00000000 (*A. rubra*), GLAV00000000 (*A. pinnata*), GLAW00000000 (*M. mutica*), and GLAX00000000 (*S. molesta*). The versions described in this paper are the first versions, GLAU01000000, GLAV01000000, GLAW01000000, and GLAX01000000. The mass spectrometry proteomics data have been deposited to the ProteomeXchange Consortium via the PRIDE (Perez-Riverol et al. 2025) partner repository with the dataset identifier PXD065205. The code used to process this data and generate the information for the tables and figures is archived at 10.5281/zenodo.14511549.

## References

[msaf241-B1] Andrés-Colás N et al Multiple PPR protein interactions are involved in the RNA editing system in *Arabidopsis* mitochondria and plastids. Proc Natl Acad Sci U S A. 2017:114:8883–8888. 10.1073/pnas.1705815114. 28761003 PMC5565447

[msaf241-B2] Aryamanesh N et al The pentatricopeptide repeat protein EMB2654 is essential for trans-splicing of a chloroplast small ribosomal subunit transcript. Plant Physiol. 2017:173:1164–1176. 10.1104/pp.16.01840. 28011633 PMC5291019

[msaf241-B3] Bankevich A et al SPAdes: a new genome assembly algorithm and its applications to single-cell sequencing. J Comput Biol. 2012:19:455–477. 10.1089/cmb.2012.0021. 22506599 PMC3342519

[msaf241-B4] Berrissou C et al Extensive import of nucleus-encoded tRNAs into chloroplasts of the photosynthetic lycophyte, *Selaginella kraussiana*. Proc Natl Acad Sci U S A. 2024:121:e2412221121. 10.1073/pnas.2412221121. 39503889 PMC11573648

[msaf241-B5] Binder S, Marchfelder A, Brennicke A. RNA editing of tRNA(Phe) and tRNA(Cys) in mitochondria of Oenothera berteriana is initiated in precursor molecules. Mol Gen Genet. 1994:244:67–74. 10.1007/BF00280188. 8041363

[msaf241-B6] Binder S, Stoll K, Stoll B. P-class pentatricopeptide repeat proteins are required for efficient 5′ end formation of plant mitochondrial transcripts. RNA Biol. 2013:10:1511–1519. 10.4161/rna.26129. PMC3858434 24184847

[msaf241-B7] Blocker FJH et al Domain structure and three-dimensional model of a group II intron-encoded reverse transcriptase. RNA. 2005:11:14–28. 10.1261/rna.7181105. 15574519 PMC1370687

[msaf241-B8] Boussardon C et al Two interacting proteins are necessary for the editing of the NdhD-1 site in *Arabidopsis* plastids. Plant Cell. 2012:24:3684–3694. 10.1105/tpc.112.099507. 23001034 PMC3480295

[msaf241-B9] Budar F, Touzet P, De Paepe R. The nucleo-mitochondrial conflict in cytoplasmic male sterilities revisited. Genetica. 2003:117:3–16. 10.1023/A:1022381016145. 12656568

[msaf241-B10] Bushmanova E, Antipov D, Lapidus A, Prjibelski AD. rnaSPAdes: a *de novo* transcriptome assembler and its application to RNA-Seq data. Gigascience. 2019:8:giz100. 10.1093/gigascience/giz100. 31494669 PMC6736328

[msaf241-B11] Bushnell B, Rood J, Singer E. BBMerge - accurate paired shotgun read merging via overlap. PLoS One. 2017:12:e0185056. 29073143 10.1371/journal.pone.0185056 PMC5657622

[msaf241-B12] Castandet B, Araya A. The nucleocytoplasmic conflict, a driving force for the emergence of plant organellar RNA editing. IUBMB Life. 2012:64:120–125. 10.1002/iub.581. 22162179

[msaf241-B13] Castandet B, Choury D, Bégu D, Jordana X, Araya A. Intron RNA editing is essential for splicing in plant mitochondria. Nucleic Acids Res. 2010:38:7112–7121. 10.1093/nar/gkq591. 20615898 PMC2978366

[msaf241-B14] Chalk AM, Taylor S, Heraud-Farlow JE, Walkley CR. The majority of A-to-I RNA editing is not required for mammalian homeostasis. Genome Biol. 2019:20:268. 10.1186/s13059-019-1873-2. 31815657 PMC6900863

[msaf241-B15] Chambers MC et al A cross-platform toolkit for mass spectrometry and proteomics. Nat Biotechnol. 2012:30:918–920. 10.1038/nbt.2377. 23051804 PMC3471674

[msaf241-B16] Chan PP, Lin BY, Mak AJ, Lowe TM. tRNAscan-SE 2.0: improved detection and functional classification of transfer RNA genes. Nucleic Acids Res. 2021:49:9077–9096. 10.1093/nar/gkab688. 34417604 PMC8450103

[msaf241-B17] Chateigner-Boutin A-L, Small I. Plant RNA editing. RNA Biol. 2010:7:213–219. 10.4161/rna.7.2.11343. 20473038

[msaf241-B18] Cheng S et al Redefining the structural motifs that determine RNA binding and RNA editing by pentatricopeptide repeat proteins in land plants. Plant J. 2016:85:532–547. 10.1111/tpj.13121. 26764122

[msaf241-B19] Chun SO, Garcia ET, Rejas M, Hayes ML. A conserved lysine in an ion-pair with a catalytic glutamate is critical for U-to-C RNA editing but restricts C-to-U RNA editing. Biochemistry. 2025:64:15–19. 10.1021/acs.biochem.4c00625. 39653594 PMC11713852

[msaf241-B20] Cognat V, Pawlak G, Pflieger D, Drouard L. PlantRNA 2.0: an updated database dedicated to tRNAs of photosynthetic eukaryotes. Plant J. 2022:112:1112–1119. 10.1111/tpj.15997. 36196656

[msaf241-B21] Covello PS, Gray MW. RNA editing in plant mitochondria. Nature. 1989:341:662–666. 10.1038/341662a0. 2552326

[msaf241-B22] Covello PS, Gray MW. On the evolution of RNA editing. Trends Genet. 1993:9:265–268. 10.1016/0168-9525(93)90011-6. 8379005

[msaf241-B23] Crooks GE, Hon G, Chandonia J-M, Brenner SE. WebLogo: a sequence logo generator. Genome Res. 2004:14:1188–1190. 10.1101/gr.849004. 15173120 PMC419797

[msaf241-B24] Diaz MF, Bentolila S, Hayes ML, Hanson MR, Mulligan RM. A protein with an unusually short PPR domain, MEF8, affects editing at over 60 Arabidopsis mitochondrial C targets of RNA editing. Plant J. 2017:92:638–649. 10.1111/tpj.13709. 29035004

[msaf241-B25] Dierckxsens N, Mardulyn P, Smits G. NOVOPlasty: *de novo* assembly of organelle genomes from whole genome data. Nucleic Acids Res. 2016:45:gkw955. 10.1093/nar/gkw955. PMC5389512 28204566

[msaf241-B26] Duan Y, Dou S, Luo S, Zhang H, Lu J. Adaptation of A-to-I RNA editing in *Drosophila*. PLoS Genet. 2017:13:e1006648. 10.1371/journal.pgen.1006648. 28282384 PMC5365144

[msaf241-B27] Duncan O, Trösch J, Fenske R, Taylor NL, Millar AH. Resource: mapping the *Triticum aestivum* proteome. Plant J. 2017:89:601–616. 10.1111/tpj.13402. 27775198

[msaf241-B28] Eisenberg E, Levanon EY. A-to-I RNA editing—immune protector and transcriptome diversifier. Nat RevGenet. 2018:19:473–490. 10.1038/s41576-018-0006-1. 29692414

[msaf241-B29] Eng JK, Jahan TA, Hoopmann MR. Comet: an open-source MS/MS sequence database search tool. Proteomics. 2013:13:22–24. 10.1002/pmic.201200439. 23148064

[msaf241-B30] Fauskee BD, Kuo L-Y, Heath TA, Xie P-J, Pryer KM. Comparative phylogenetic analyses of RNA editing in fern plastomes suggest possible adaptive innovations. New Phytol. 2025:247:2945–2963. 10.1111/nph.70244. 40415590

[msaf241-B31] Fauskee BD, Sigel EM, Pryer KM, Grusz AL. Variation in frequency of plastid RNA editing within *Adiantum* implies rapid evolution in fern plastomes. Am J Bot. 2021:108:820–827. 10.1002/ajb2.1649. 33969475

[msaf241-B32] Feng Y, Wicke S. New mitochondrial genomes of leptosporangiate ferns allow modeling the mitogenomic inflation syndrome across all land plant lineages. bioRxiv 521604. 10.1101/2022.12.23.521604, 23 December 2022, preprint: not peer reviewed.

[msaf241-B33] Freyer R, Kiefer-Meyer MC, Kössel H. Occurrence of plastid RNA editing in all major lineages of land plants. Proc Natl Acad Sci U S A. 1997:94:6285–6290. 10.1073/pnas.94.12.6285. 9177209 PMC21041

[msaf241-B34] Fujii S, Small I. The evolution of RNA editing and pentatricopeptide repeat genes: tansley review. New Phytol. 2011:191:37–47. 10.1111/j.1469-8137.2011.03746.x. 21557747

[msaf241-B35] Gao L, Yi X, Yang Y-X, Su Y-J, Wang T. Complete chloroplast genome sequence of a tree fern Alsophila spinulosa: insights into evolutionary changes in fern chloroplast genomes. BMC Evol Biol. 2009:9:130. 10.1186/1471-2148-9-130. 19519899 PMC2706227

[msaf241-B36] Gerke P et al Towards a plant model for enigmatic U-to-C RNA editing: the organelle genomes, transcriptomes, editomes and candidate RNA editing factors in the hornwort *Anthoceros agrestis*. New Phytol. 2020:225:1974–1992. 10.1111/nph.16297. 31667843

[msaf241-B37] Giegé P, Brennicke A. RNA editing in *Arabidopsis* mitochondria effects 441 C to U changes in ORFs. Proc Natl Acad Sci U S A. 1999:96:15324–15329. 10.1073/pnas.96.26.15324. 10611383 PMC24818

[msaf241-B38] Gobert A et al A single Arabidopsis organellar protein has RNase P activity. Nat Struct Mol Biol. 2010:17:740–744. 10.1038/nsmb.1812. 20473316

[msaf241-B39] Gray MW . Evolutionary origin of RNA editing. Biochemistry. 2012:51:5235–5242. 10.1021/bi300419r. 22708551

[msaf241-B40] Gray MW, Covello PS. RNA editing in plant mitochondria and chloroplasts. FASEB J. 1993:7:64–71. 10.1096/fasebj.7.1.8422976. 8422976

[msaf241-B41] Grewe F et al A unique transcriptome: 1782 positions of RNA editing alter 1406 codon identities in mitochondrial mRNAs of the lycophyte *Isoetes engelmannii*. Nucleic Acids Res. 2011:39:2890–2902. 10.1093/nar/gkq1227. 21138958 PMC3074146

[msaf241-B42] Gualberto JM, Weil JH, Grienenberger JM. Editing of the wheat *cox*III transcript: evidence for twelve C to U and one U to C conversions and for sequence similarities around editing sites. Nucleic Acids Res. 1990:18:3771–3776. 10.1093/nar/18.13.3771. 1695731 PMC331076

[msaf241-B43] Guo W, Grewe F, Mower JP. Variable frequency of plastid RNA editing among ferns and repeated loss of uridine-to-cytidine editing from vascular plants. PLoS One. 2015:10:e0117075. 10.1371/journal.pone.0117075. 25568947 PMC4287625

[msaf241-B44] Guo W, Zhu A, Fan W, Mower JP. Complete mitochondrial genomes from the ferns *Ophioglossum californicum* and *Psilotum nudum* are highly repetitive with the largest organellar introns. New Phytol. 2017:213:391–403. 10.1111/nph.14135. 27539928

[msaf241-B45] Gutmann B et al The expansion and diversification of pentatricopeptide repeat RNA-editing factors in plants. Mol Plant. 2020:13:215–230. 10.1016/j.molp.2019.11.002. 31760160

[msaf241-B46] Hao J, Liang Y, Su Y, Wang T. The complete mitochondrial genome of Ophioglossum vulgatum L. is with highly repetitive sequences: intergenomic fragment transfer and phylogenetic analysis. Genes (Basel). 2022:13:1287. 10.3390/genes13071287. 35886070 PMC9316493

[msaf241-B47] Hayes ML, Garcia ET, Chun SO, Selke M. Crosslinking of base-modified RNAs by synthetic DYW-KP base editors implicates an enzymatic lysine as the nitrogen donor for U-to-C RNA editing. J Biol Chem. 2024:300:107454. 10.1016/j.jbc.2024.107454. 38852885 PMC11332814

[msaf241-B48] Hayes ML, Santibanez PI. A plant pentatricopeptide repeat protein with a DYW-deaminase domain is sufficient for catalyzing C-to-U RNA editing in vitro. J Biol Chem. 2020:295:3497–3505. 10.1074/jbc.RA119.011790. 31996373 PMC7076202

[msaf241-B49] Hecht J, Grewe F, Knoop V. Extreme RNA editing in coding islands and abundant microsatellites in repeat sequences of *Selaginella moellendorffii* mitochondria: the root of frequent plant mtDNA recombination in early tracheophytes. Genome Biol Evol. 2011:3:344–358. 10.1093/gbe/evr027. 21436122 PMC5654404

[msaf241-B50] Hegeman CE, Hayes ML, Hanson MR. Substrate and cofactor requirements for RNA editing of chloroplast transcripts in Arabidopsis *in vitro*. Plant J. 2005:42:124–132. 10.1111/j.1365-313X.2005.02360.x. 15773858

[msaf241-B51] Hiesel R, Wissinger B, Schuster W, Brennicke A. RNA editing in plant mitochondria. Science. 1989:246:1632–1634. 10.1126/science.2480644. 2480644

[msaf241-B52] Howad W, Kempken F. Cell type-specific loss of *atp6* RNA editing in cytoplasmic male sterile *Sorghum bicolor*. Proc Natl Acad Sci U S A. 1997:94:11090–11095. 10.1073/pnas.94.20.11090. 9380764 PMC23623

[msaf241-B53] Huynh SD, Melonek J, Colas des Francs-Small C, Bond CS, Small I. A unique C-terminal domain contributes to the molecular function of restorer-of-fertility proteins in plant mitochondria. New Phytol. 2023:240:830–845. 10.1111/nph.19166. 37551058

[msaf241-B54] Ichinose M et al U-to-C RNA editing by synthetic PPR-DYW proteins in bacteria and human culture cells. Commun Biol. 2022:5:968. 10.1038/s42003-022-03927-3. 36109586 PMC9478123

[msaf241-B55] Ichinose M et al Fine-tuning of the PPR protein directs the RNA editing activity toward C-to-U or U-to-C conversion. Sci Rep. 2025:15:6288. 10.1038/s41598-025-90722-9. 39984571 PMC11845758

[msaf241-B56] Ichinose M, Sugita M. RNA editing and its molecular mechanism in plant organelles. Genes (Basel). 2016:8:5. 10.3390/genes8010005. 28025543 PMC5295000

[msaf241-B57] Ishibashi K, Small I, Shikanai T. Evolutionary model of plastidial RNA editing in angiosperms presumed from genome-wide analysis of Amborella trichopoda. Plant Cell Physiol. 2019:60:2141–2151. 10.1093/pcp/pcz111. 31150097

[msaf241-B58] Jiang D, Zhang J. The preponderance of nonsynonymous A-to-I RNA editing in coleoids is nonadaptive. Nat Commun. 2019:10:5411. 10.1038/s41467-019-13275-2. 31776345 PMC6881472

[msaf241-B59] Jin J-J et al GetOrganelle: a fast and versatile toolkit for accurate de novo assembly of organelle genomes. Genome Biol. 2020:21:241. 10.1186/s13059-020-02154-5. 32912315 PMC7488116

[msaf241-B60] Jobson RW, Qiu Y-L. Did RNA editing in plant organellar genomes originate under natural selection or through genetic drift? Biol Direct. 2008:3:43. 10.1186/1745-6150-3-43. 18939975 PMC2584032

[msaf241-B61] Käll L, Canterbury JD, Weston J, Noble WS, MacCoss MJ. Semi-supervised learning for peptide identification from shotgun proteomics datasets. Nat Methods. 2007:4:923–925. 10.1038/nmeth1113. 17952086

[msaf241-B62] Katoh K, Standley DM. MAFFT multiple sequence alignment software version 7: improvements in performance and usability. Mol Biol Evol. 2013:30:772–780. 10.1093/molbev/mst010. 23329690 PMC3603318

[msaf241-B63] Kessner D, Chambers M, Burke R, Agus D, Mallick P. ProteoWizard: open source software for rapid proteomics tools development. Bioinformatics. 2008:24:2534–2536. 10.1093/bioinformatics/btn323. 18606607 PMC2732273

[msaf241-B64] Knie N, Grewe F, Fischer S, Knoop V. Reverse U-to-C editing exceeds C-to-U RNA editing in some ferns—a monilophyte-wide comparison of chloroplast and mitochondrial RNA editing suggests independent evolution of the two processes in both organelles. BMC Evol Biol. 2016:16:134. 10.1186/s12862-016-0707-z. 27329857 PMC4915041

[msaf241-B65] Kugita M, Yamamoto Y, Fujikawa T, Matsumoto T, Yoshinaga K. RNA editing in hornwort chloroplasts makes more than half the genes functional. Nucleic Acids Res. 2003:31:2417–2423. 10.1093/nar/gkg327. 12711687 PMC154213

[msaf241-B66] Kuznetsov D et al OrthoDB v11: annotation of orthologs in the widest sampling of organismal diversity. Nucleic Acids Res. 2023:51:D445–D451. 10.1093/nar/gkac998. 36350662 PMC9825584

[msaf241-B67] Kwok van der Giezen FM et al Insights into U-to-C RNA editing from the lycophyte *Phylloglossum drummondii*. Plant J. 2024:119:445–459. 10.1111/tpj.16775. 38652016

[msaf241-B68] Lee K et al The coordinated action of PPR4 and EMB2654 on each intron half mediates *trans*-splicing of *rps12* transcripts in plant chloroplasts. Plant J. 2019:100:1193–1207. 10.1111/tpj.14509. 31442349

[msaf241-B69] Lesch E et al Plant mitochondrial RNA editing factors can perform targeted C-to-U editing of nuclear transcripts in human cells. Nucleic Acids Res. 2022:50:9966–9983. 10.1093/nar/gkac752. 36107771 PMC9508816

[msaf241-B70] Li F-W et al Fern genomes elucidate land plant evolution and cyanobacterial symbioses. Nat Plants. 2018:4:460–472. 10.1038/s41477-018-0188-8. 29967517 PMC6786969

[msaf241-B71] Lukeš J, Archibald JM, Keeling PJ, Doolittle WF, Gray MW. How a neutral evolutionary ratchet can build cellular complexity. IUBMB Life. 2011:63:528–537. 10.1002/iub.489. 21698757

[msaf241-B72] Lurin C et al Genome-wide analysis of Arabidopsis pentatricopeptide repeat proteins reveals their essential role in organelle biogenesis. Plant Cell. 2004:16:2089–2103. 10.1105/tpc.104.022236. 15269332 PMC519200

[msaf241-B73] Manni M, Berkeley MR, Seppey M, Simão FA, Zdobnov EM. BUSCO update: novel and streamlined workflows along with broader and deeper phylogenetic coverage for scoring of eukaryotic, prokaryotic, and viral genomes. Mol Biol Evol. 2021:38:4647–4654. 10.1093/molbev/msab199. 34320186 PMC8476166

[msaf241-B74] Melonek J et al The genetic basis of cytoplasmic male sterility and fertility restoration in wheat. Nat Commun. 2021:12:1036. 10.1038/s41467-021-21225-0. 33589621 PMC7884431

[msaf241-B75] Metzgar JS, Schneider H, Pryer KM. Phylogeny and divergence time estimates for the fern Genus *Azolla* (Salviniaceae). Int J Plant Sci. 2007:168:1045–1053. 10.1086/519007.

[msaf241-B76] Miyata Y, Sugita M. Tissue- and stage-specific RNA editing of rps 14 transcripts in moss (Physcomitrella patens) chloroplasts. J Plant Physiol. 2004:161:113–115. 10.1078/0176-1617-01220. 15002671

[msaf241-B77] Mower JP . Modeling sites of RNA editing as a fifth nucleotide state reveals progressive loss of edited sites from angiosperm mitochondria. Mol Biol Evol. 2008:25:52–61. 10.1093/molbev/msm226. 17940211

[msaf241-B78] Neckermann K, Zeltz P, Igloi GL, Kössel H, Maier RM. The role of RNA editing in conservation of start codons in chloroplast genomes. Gene. 1994:146:177–182. 10.1016/0378-1119(94)90290-9. 8076816

[msaf241-B79] Oldenkott B, Yamaguchi K, Tsuji-Tsukinoki S, Knie N, Knoop V. Chloroplast RNA editing going extreme: more than 3400 events of C-to-U editing in the chloroplast transcriptome of the lycophyte *Selaginella uncinata*. RNA. 2014:20:1499–1506. 10.1261/rna.045575.114. 25142065 PMC4174432

[msaf241-B80] Oldenkott B, Yang Y, Lesch E, Knoop V, Schallenberg-Rüdinger M. Plant-type pentatricopeptide repeat proteins with a DYW domain drive C-to-U RNA editing in *Escherichia coli*. Commun Biol. 2019:2:85. 10.1038/s42003-019-0328-3. 30854477 PMC6397227

[msaf241-B81] Peng Y, Wang Z, Li M, Wang T, Su Y. Characterization and analysis of multi-organ full-length transcriptomes in Sphaeropteris brunoniana and Alsophila latebrosa highlight secondary metabolism and chloroplast RNA editing pattern of tree ferns. BMC Plant Biol. 2024:24:73. 10.1186/s12870-024-04746-w. 38273309 PMC10811885

[msaf241-B82] Perez-Riverol Y et al The PRIDE database at 20 years: 2025 update. Nucleic Acids Res. 2025:53:D543–D553. 10.1093/nar/gkae1011. 39494541 PMC11701690

[msaf241-B83] Pfalz J, Bayraktar OA, Prikryl J, Barkan A. Site-specific binding of a PPR protein defines and stabilizes 5′ and 3′ mRNA termini in chloroplasts. EMBO J. 2009:28:2042–2052. 10.1038/emboj.2009.121. 19424177 PMC2718276

[msaf241-B84] Pryer KM et al Phylogeny and evolution of ferns (monilophytes) with a focus on the early leptosporangiate divergences. Am J Bot. 2004:91:1582–1598. 10.3732/ajb.91.10.1582. 21652310

[msaf241-B85] Qi Z et al Adaptive advantages of restorative RNA editing in fungi for resolving survival-reproduction trade-offs. Sci Adv. 2024:10:eadk6130. 10.1126/sciadv.adk6130. 38181075 PMC10776026

[msaf241-B86] Robison TA et al Mobile elements shape plastome evolution in ferns. Genome Biol Evol. 2018:10:2558–2571. 10.1093/gbe/evy189. 30165616 PMC6166771

[msaf241-B87] Rüdinger M, Funk HT, Rensing SA, Maier UG, Knoop V. RNA editing: only eleven sites are present in the Physcomitrella patens mitochondrial transcriptome and a universal nomenclature proposal. Mol Genet Genomics. 2009:281:473–481. 10.1007/s00438-009-0424-z. 19169711

[msaf241-B88] Rüdinger M, Polsakiewicz M, Knoop V. Organellar RNA editing and plant-specific extensions of pentatricopeptide repeat proteins in jungermanniid but not in marchantiid liverworts. Mol Biol Evol. 2008:25:1405–1414. 10.1093/molbev/msn084. 18400790

[msaf241-B89] Rugen N, Senkler M, Braun H-P. Deep proteomics reveals incorporation of unedited proteins into mitochondrial protein complexes in Arabidopsis. Plant Physiol. 2024:195:1180–1199. 10.1093/plphys/kiad655. 38060994 PMC11142381

[msaf241-B90] Ruwe H, Schmitz-Linneweber C. Short non-coding RNA fragments accumulating in chloroplasts: footprints of RNA binding proteins? Nucleic Acids Res. 2012:40:3106–3116. 10.1093/nar/gkr1138. 22139936 PMC3326302

[msaf241-B91] Sale PJM, Orr PT, Shell GS, Erskine DJC. Photosynthesis and growth rates in Salvinia molesta and Eichhornia crassipes. J Appl Ecol. 1985:22:125. 10.2307/2403332.

[msaf241-B92] Salone V et al A hypothesis on the identification of the editing enzyme in plant organelles. FEBS Lett. 2007:581:4132–4138. 10.1016/j.febslet.2007.07.075. 17707818

[msaf241-B93] San Filippo J, Lambowitz AM. Characterization of the C-terminal DNA-binding/DNA endonuclease region of a group II intron-encoded protein. J Mol Biol. 2002:324:933–951. 10.1016/S0022-2836(02)01147-6. 12470950

[msaf241-B94] Schmitz-Linneweber C et al A pentatricopeptide repeat protein facilitates the *trans*-splicing of the maize chloroplast *rps12* pre-mRNA. Plant Cell. 2006:18:2650–2663. 10.1105/tpc.106.046110. 17041147 PMC1626628

[msaf241-B95] Sigel EM et al Being ghosted: determining the progenitor genomes and biogeographic origin of the invasive fern giant salvinia. Biol Invasions. 2025:27:1–15. 10.1007/s10530-025-03580-x.

[msaf241-B96] Simão FA, Waterhouse RM, Ioannidis P, Kriventseva EV, Zdobnov EM. BUSCO: assessing genome assembly and annotation completeness with single-copy orthologs. Bioinformatics. 2015:31:3210–3212. 10.1093/bioinformatics/btv351. 26059717

[msaf241-B97] Sloan DB . Can transcriptome size and off-target effects explain the contrasting evolution of mitochondrial vs nuclear RNA editing? J Evol Biol. 2025:voaf042. 10.1093/jeb/voaf042. 40323724 PMC12710240

[msaf241-B98] Sloan DB, MacQueen AH, Alverson AJ, Palmer JD, Taylor DR. Extensive loss of RNA editing sites in rapidly evolving Silene mitochondrial genomes: selection vs. retroprocessing as the driving force. Genetics. 2010:185:1369–1380. 10.1534/genetics.110.118000. 20479143 PMC2927763

[msaf241-B99] Smith DR . Unparalleled variation in RNA editing among *Selaginella* plastomes. Plant Physiol. 2020:182:12–14. 10.1104/pp.19.00904. 31481629 PMC6945854

[msaf241-B100] Song M et al A novel chloroplast gene reported for flagellate plants. Am J Bot. 2018:105:117–121. 10.1002/ajb2.1010. 29532931

[msaf241-B101] Song Y-Y et al The complete mitochondrial genome of *Dryopteris crassirhizoma* Nakai (Dryopteridaceae, *Dryopteris* Adanson). Mitochondrial DNA B Resour. 2021:6:2704–2705. 10.1080/23802359.2021.1966344. 34435126 PMC8381936

[msaf241-B102] Steinhauser S, Beckert S, Capesius I, Malek O, Knoop V. Plant mitochondrial RNA editing. J Mol Evol. 1999:48:303–312. 10.1007/PL00006473. 10093219

[msaf241-B103] Takenaka M et al DYW domain structures imply an unusual regulation principle in plant organellar RNA editing catalysis. Nat Catal. 2021:4:510–522. 10.1038/s41929-021-00633-x. 34712911 PMC7611903

[msaf241-B104] Testo W, Sundue M. A 4000-species dataset provides new insight into the evolution of ferns. Mol Phylogenet Evol. 2016:105:200–211. 10.1016/j.ympev.2016.09.003. 27621129

[msaf241-B105] Thielen M, Gärtner B, Knoop V, Schallenberg-Rüdinger M, Lesch E. Conquering new grounds: plant organellar C-to-U RNA editing factors can be functional in the plant cytosol. Plant J. 2024:119:895–915. 10.1111/tpj.16804. 38753873

[msaf241-B106] Träger C et al Evolution from the prokaryotic to the higher plant chloroplast signal recognition particle: the signal recognition particle RNA is conserved in plastids of a wide range of photosynthetic organisms. Plant Cell. 2013:24:4819–4836. 10.1105/tpc.112.102996. PMC3556960 23275580

[msaf241-B107] van Wijk KJ et al Detection and editing of the updated Arabidopsis plastid- and mitochondrial-encoded proteomes through PeptideAtlas. Plant Physiol. 2024:194:1411–1430. 10.1093/plphys/kiad572. 37879112

[msaf241-B108] Wagner GM . Azolla: a review of its biology and utilization. Bot Rev. 1997:63:1–26. 10.1007/BF02857915.

[msaf241-B109] Walker BJ et al Pilon: an integrated tool for comprehensive microbial variant detection and genome assembly improvement. PLoS One. 2014:9:e112963. 10.1371/journal.pone.0112963. 25409509 PMC4237348

[msaf241-B110] Waneka G et al Disruption of recombination machinery alters the mutational landscape in plant organellar genomes. G3 (Bethesda). 2025:15:jkaf029. 10.1093/g3journal/jkaf029. 39946260 PMC12005158

[msaf241-B111] Wick RR, Schultz MB, Zobel J, Holt KE. Bandage: interactive visualization of *de novo* genome assemblies. Bioinformatics. 2015:31:3350–3352. 10.1093/bioinformatics/btv383. 26099265 PMC4595904

[msaf241-B112] Wolf PG, Rowe CA, Hasebe M. High levels of RNA editing in a vascular plant chloroplast genome: analysis of transcripts from the fern Adiantum capillus-veneris. Gene. 2004:339:89–97. 10.1016/j.gene.2004.06.018. 15363849

[msaf241-B113] Wolf PG, Rowe CA, Sinclair RB, Hasebe M. Complete nucleotide sequence of the chloroplast genome from a leptosporangiate fern, Adiantum capillus-veneris L. DNA Res. 2003:10:59–65. 10.1093/dnares/10.2.59. 12755170

[msaf241-B114] Wright AL et al The Q/R editing site of AMPA receptor GluA2 subunit acts as an epigenetic switch regulating dendritic spines, neurodegeneration and cognitive deficits in Alzheimer's disease. Mol Neurodegener. 2023:18:65. 10.1186/s13024-023-00632-5. 37759260 PMC10537207

[msaf241-B115] Wu C-S, Chaw S-M. Evolution of mitochondrial RNA editing in extant gymnosperms. Plant J. 2022:111:1676–1687. 10.1111/tpj.15916. 35877596 PMC9545813

[msaf241-B116] Xin K et al Navigating trade-offs: the adaptive significance of A-to-I RNA editing in fungi, bacteria, and animals. Epigenet Insights. 2025:18:e002. 10.48130/epi-0025-0002.

[msaf241-B117] Yoshinaga K, Iinuma H, Masuzawa T, Uedal K. Extensive RNA editing of U to C in addition to C to U substitution in the *rbcL* transcripts of hornwort chloroplasts and the origin of RNA editing in green plants. Nucleic Acids Res. 1996:24:1008–1014. 10.1093/nar/24.6.1008. 8604330 PMC145765

[msaf241-B118] Zhelyazkova P et al Protein-mediated protection as the predominant mechanism for defining processed mRNA termini in land plant chloroplasts. Nucleic Acids Res. 2012:40:3092–3105. 10.1093/nar/gkr1137. 22156165 PMC3326301

[msaf241-B119] Zhou W et al PPR-SMR protein SOT1 has RNA endonuclease activity. Proc Natl Acad Sci U S A. 2017:114:E1554–E1563. 10.1073/pnas.1612460114. 28167782 PMC5338415

[msaf241-B120] Zumkeller S, Gerke P, Knoop V. A functional twintron, “zombie” twintrons and a hypermobile group II intron invading itself in plant mitochondria. Nucleic Acids Res. 2020:48:2661–2675. 10.1093/nar/gkz1194. 31915815 PMC7049729

[msaf241-B121] Zumkeller S, Knoop V. Categorizing 161 plant (streptophyte) mitochondrial group II introns into 29 families of related paralogues finds only limited links between intron mobility and intron-borne maturases. BMC Ecol Evol. 2023:23:5. 10.1186/s12862-023-02108-y. 36915058 PMC10012718

[msaf241-B122] Zumkeller S, Polsakiewicz M, Knoop V. Rickettsial DNA and a trans-splicing rRNA group I intron in the unorthodox mitogenome of the fern Haplopteris ensiformis. Commun Biol. 2023:6:296. 10.1038/s42003-023-04659-8. 36941328 PMC10027690

