# Supplementary material for: High Conservation of Translation-Enabling RNA Editing Sites in Hyper-editing Ferns Implies They Are Not Selectively Neutral
Source: Mol Biol Evol. 2025 Sep 30;42(10):msaf241. doi: 10.1093/molbev/msaf241 (PMC12548569; doi:10.1093/molbev/msaf241)
Supplement: msaf241_Supplementary_Data [file msaf241_supplementary_data.zip › Supplemenary_Figures.pdf]

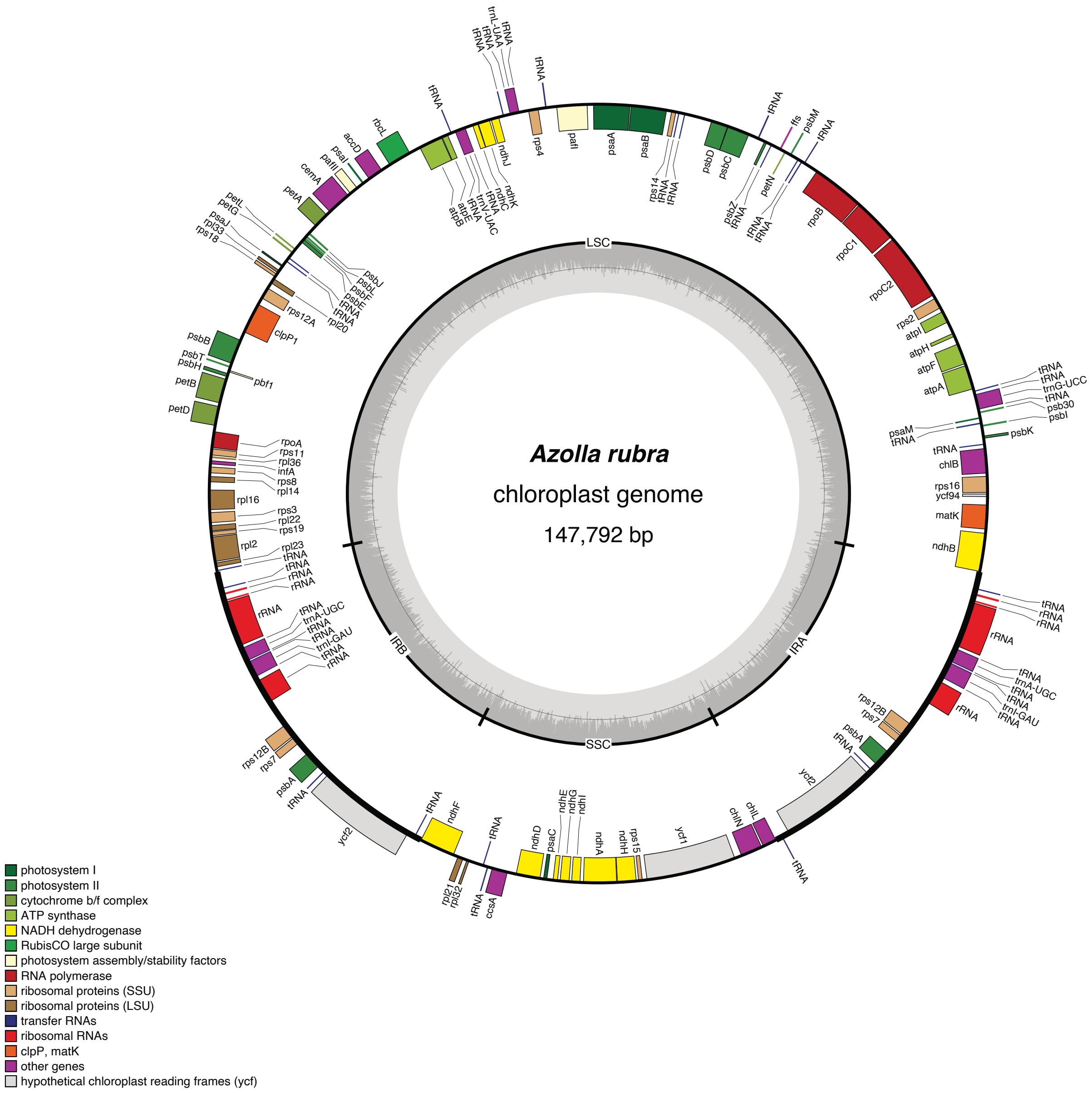

Fig. S1. The chloroplast genome of *Azolla rubra* (PQ616047) drawn by OGDraw (Greiner et al. 2019)**.**

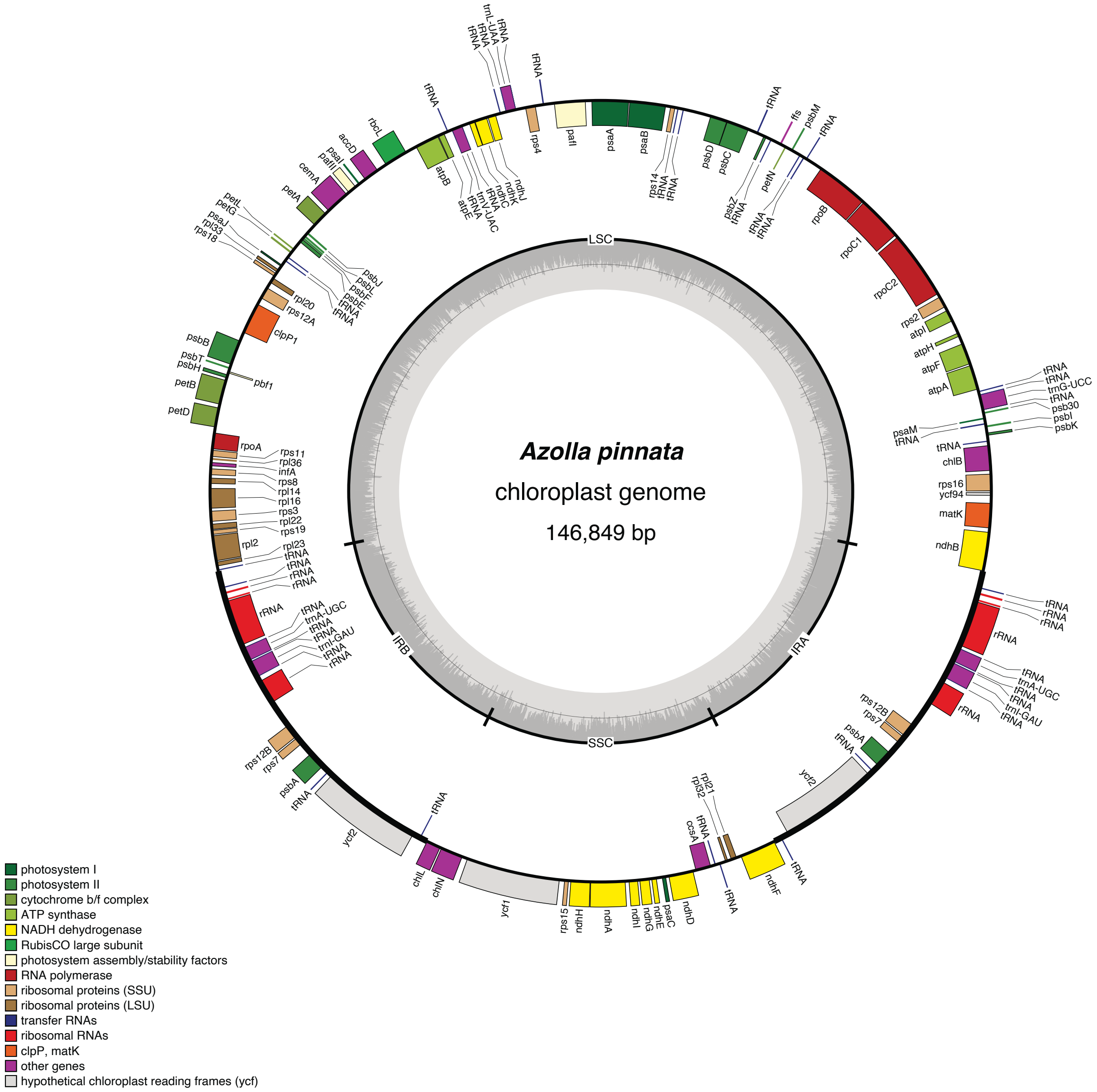

Fig. S2. The chloroplast genome of *Azolla pinnata* (PQ616048) drawn by OGDraw (Greiner et al. 2019)**.**

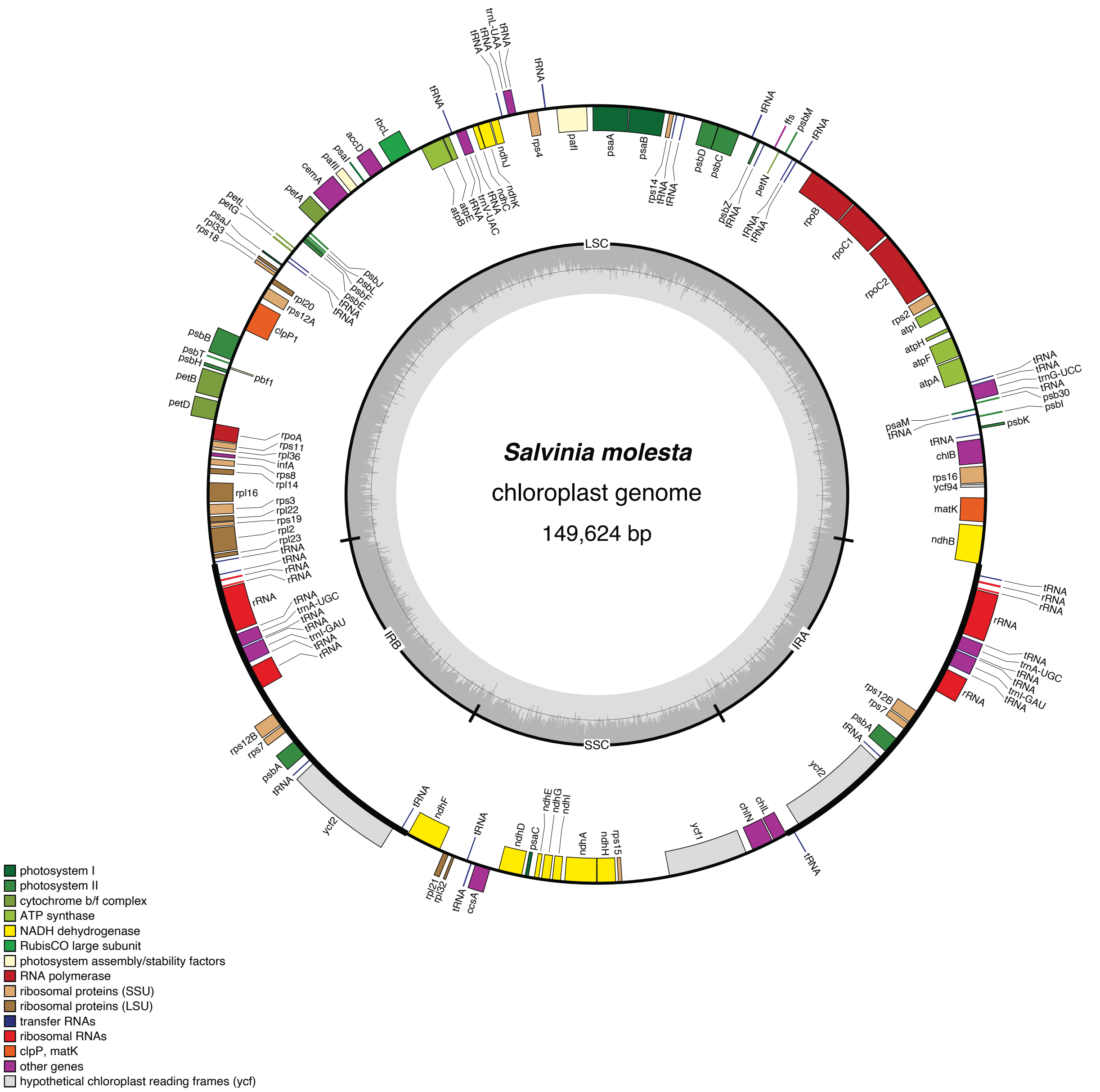

Fig. S3. The chloroplast genome of *Marsilea mutica* (PQ616049) drawn by OGDraw (Greiner et al. 2019)**.**

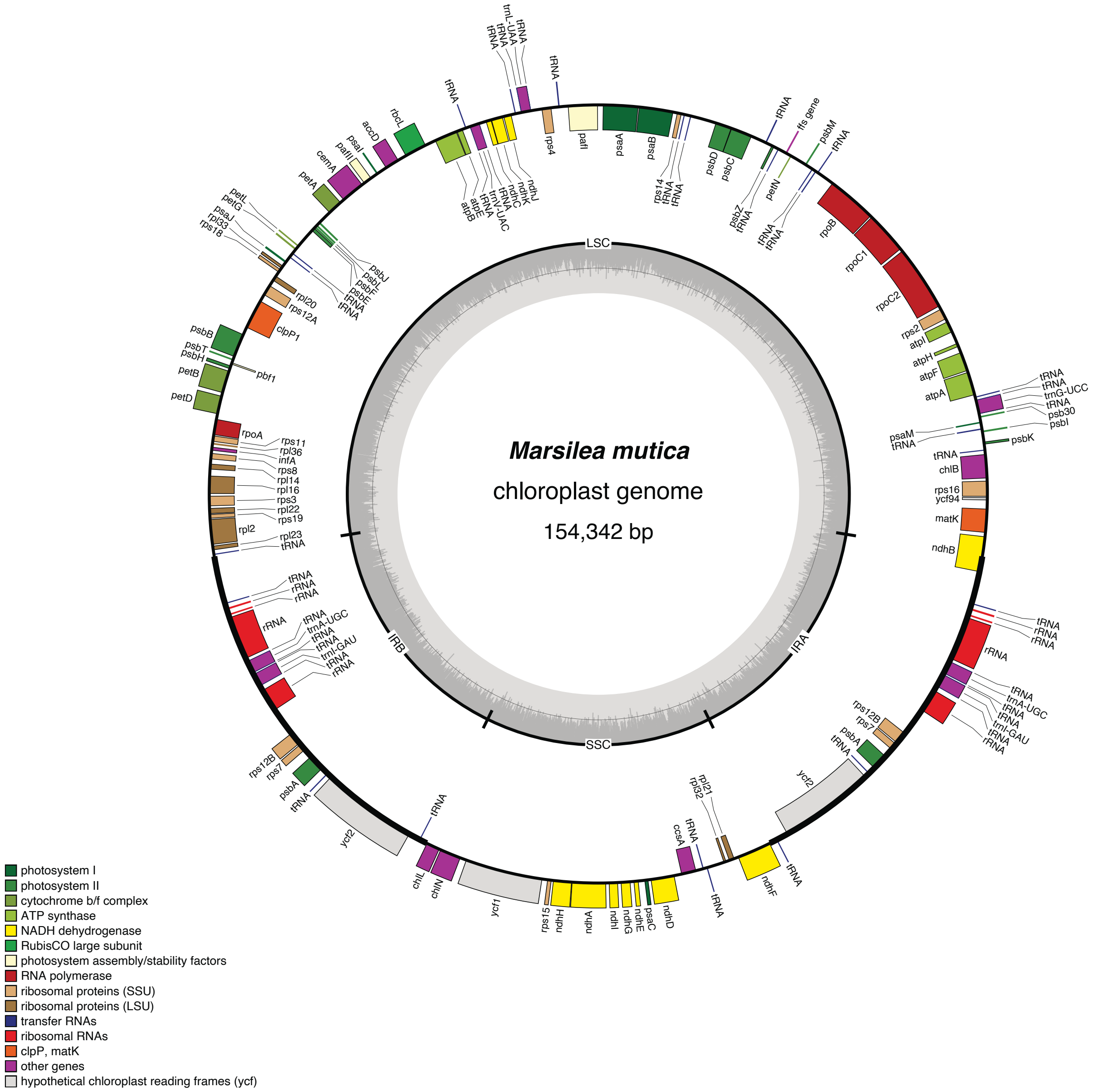

Fig. S4. The chloroplast genome of *Salvinia molesta* (PQ616050) drawn by OGDraw (Greiner et al. 2019)**.**

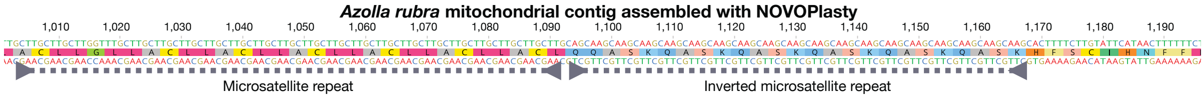

Fig. S5. Extensive microsatellite repeats in mitochondrial assemblies of *Azolla rubra*. Arrays of a short repeat form repeat regions which can confound de Bruijn graph assembly methods like NOVOPlasty and SPAdes when long stretches of the repeat region can assemble infinitely.

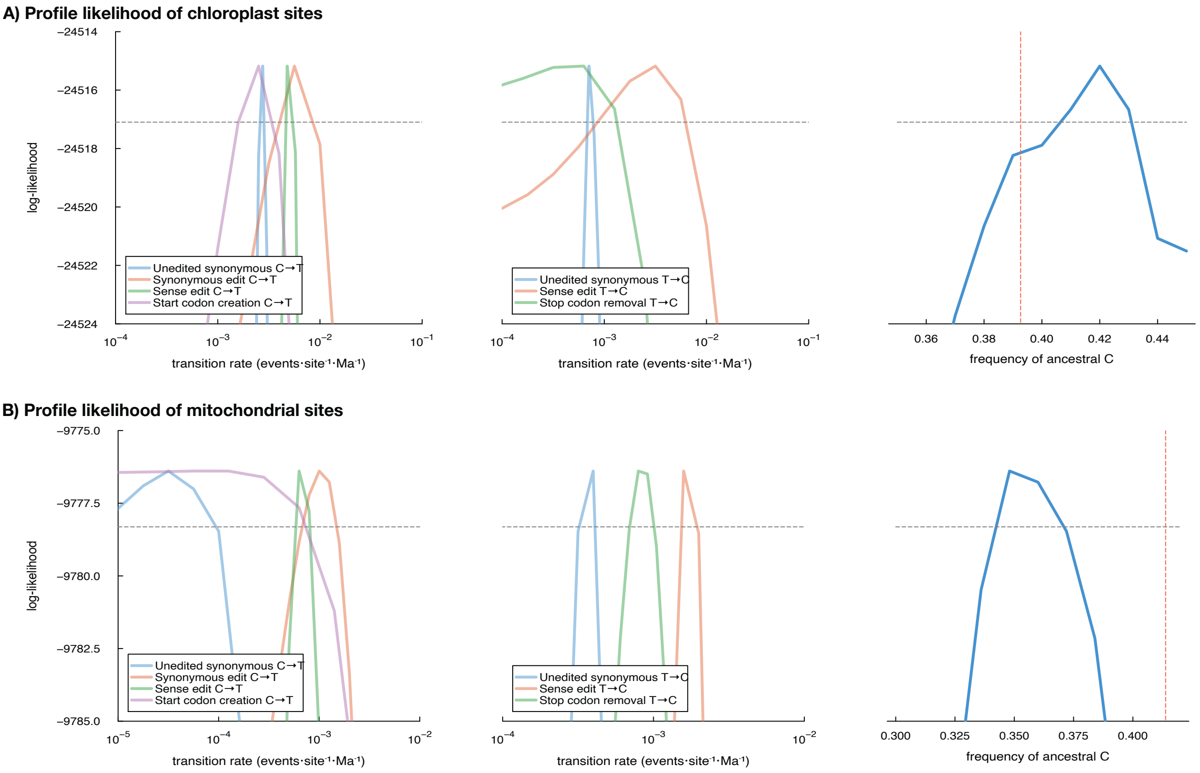

Fig. S6. Profile likelihoods of transition rates and ancestral state. A) Profile likelihoods for chloroplast parameters. Profile likelihoods show the maximum likelihood values for specific parameters when the parameter is held to a particular value and the other parameters are allowed to vary. Likelihoods are plotted as log-likelihoods. The horizontal dotted line indicates the 95% confidence threshold, i.e. the 95% confidence interval for each parameter extends between the points where the profile likelihood for that parameter intersects this line. In the plot of the profile likelihood for the ancestral state, the vertical dashed red line indicates the current frequency of C across all these sites. **B)** Profile likelihoods for mitochondrial parameters.

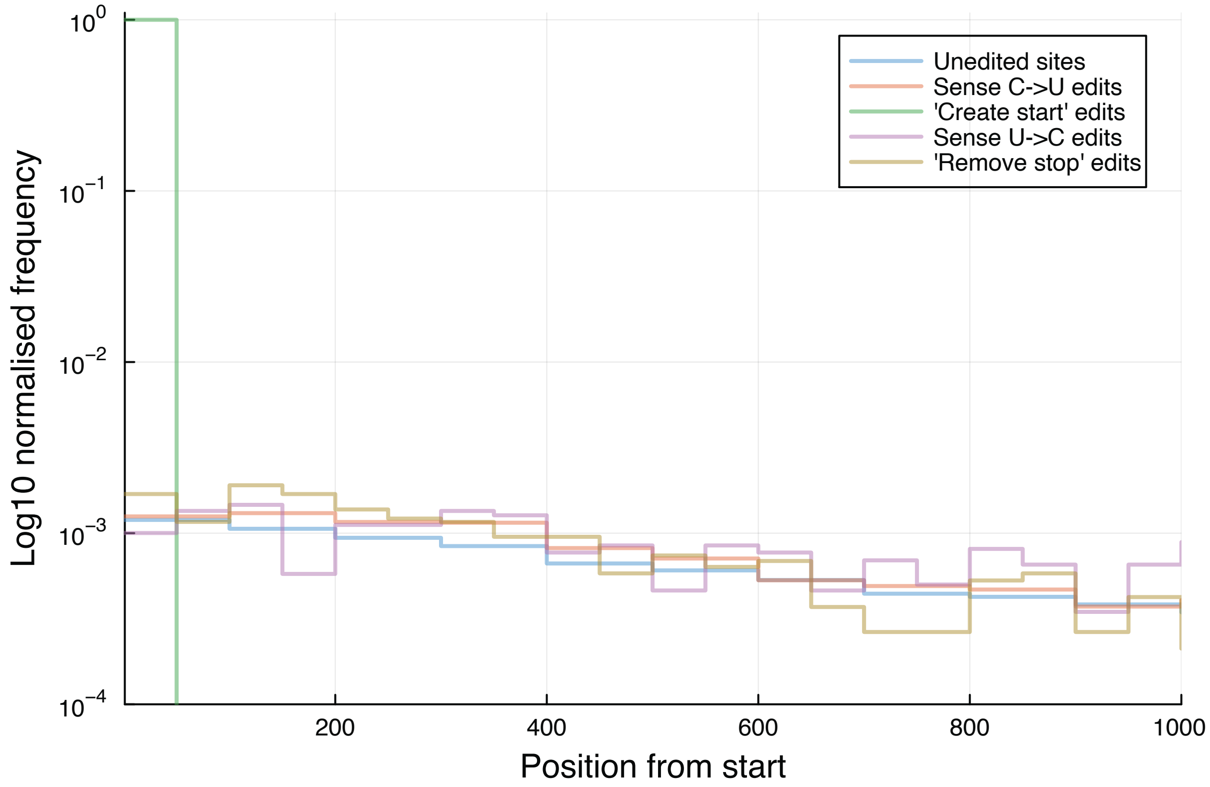

Fig. S7. Normalised frequency of RNA editing sites relative to start position in Salviniales chloroplasts and mitochondria. Histogram of normalised frequencies in protein-coding regions are from chloroplast and mitochondrial sites in the Salviniales ferns Azolla filiculoides, Azolla rubra, Azolla pinnata, Salvinia molesta and Marsilea mutica, and include 16932 unedited sites, 1772 C-to-U sense edits, 57 C-to-U create start edits, 519 U-to-C sense edits and 378 U-to-C remove stop edits.

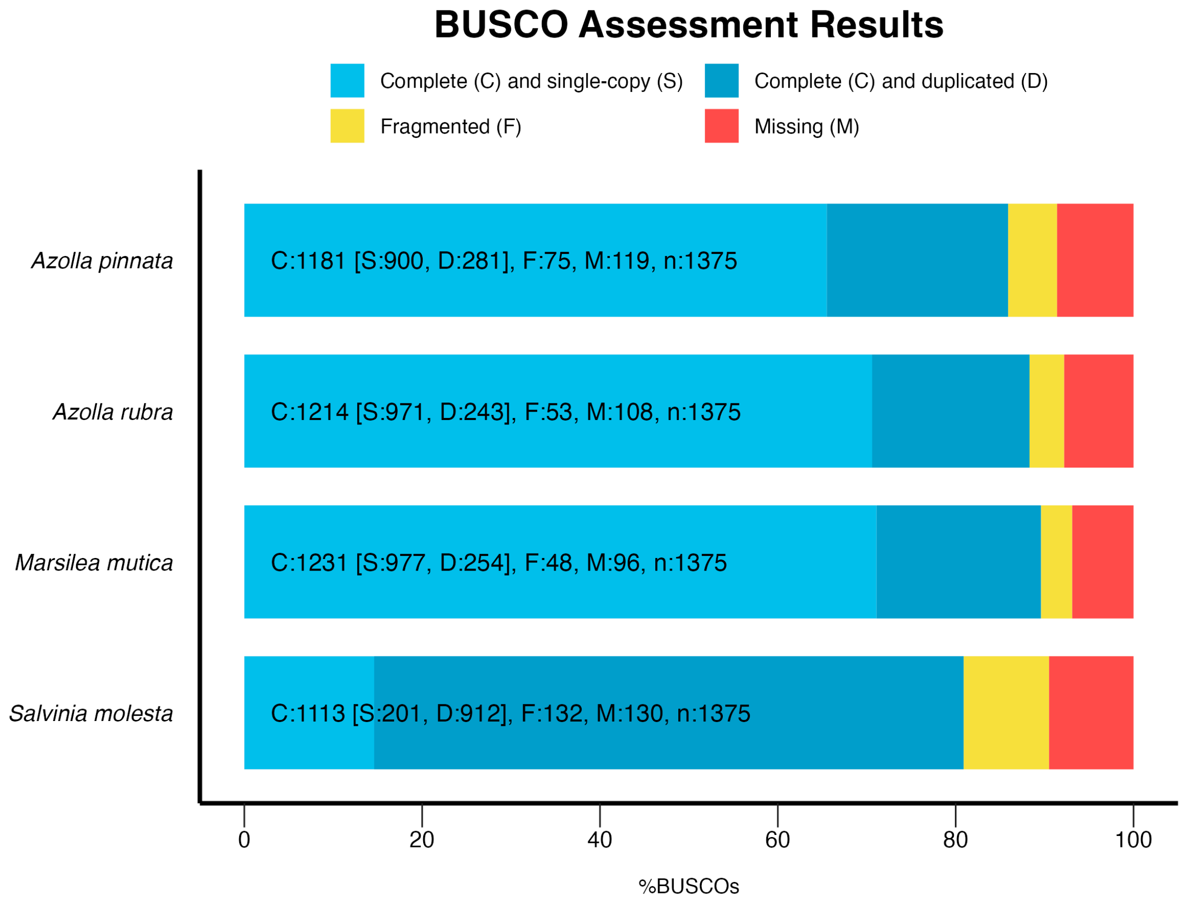

Fig. S8. Benchmarking universal single copy orthologs (BUSCO) (Simão et al. 2015; Manni et al. 2021) protein analysis of translated open reading frames generated from *de novo* transcriptome assemblies for *Salviniales* species. Protein sequences were assessed against the embryophyta_odb10 database (Kuznetsov et al. 2023) and indicate transcriptome completeness of at least 80%. The overlap of missing BUSCOs suggests that some sequences may be absent from leptosporangiate ferns, and completeness may be underestimated. The quantity of duplicated BUSCOs in *Salvinia molesta* is strongly indicative of a whole genome duplication.

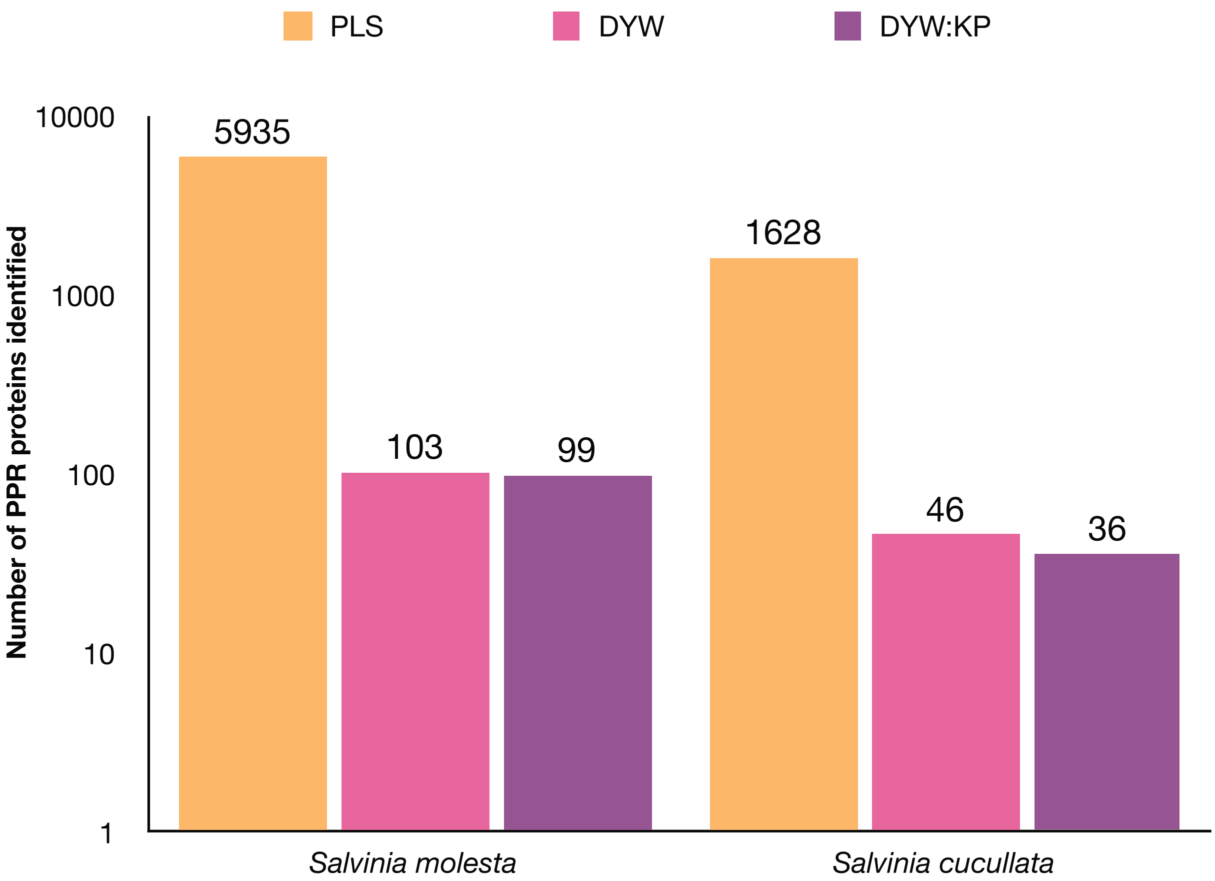

Fig. S9. Comparison of PPR proteins identified in the transcriptome of *Salvinia molesta* and the nuclear genome assembly of *Salvinia cucullata*. The abundance of PPR protein sequences in *Salvinia molesta* is most likely due to a whole genome duplication event. The number of PPR proteins identified in *Salvinia cucullata* is more similar to the estimates from *Azolla filiculoides*, *Azolla rubra*, *Azolla pinnata* and *Marsilea mutica*.

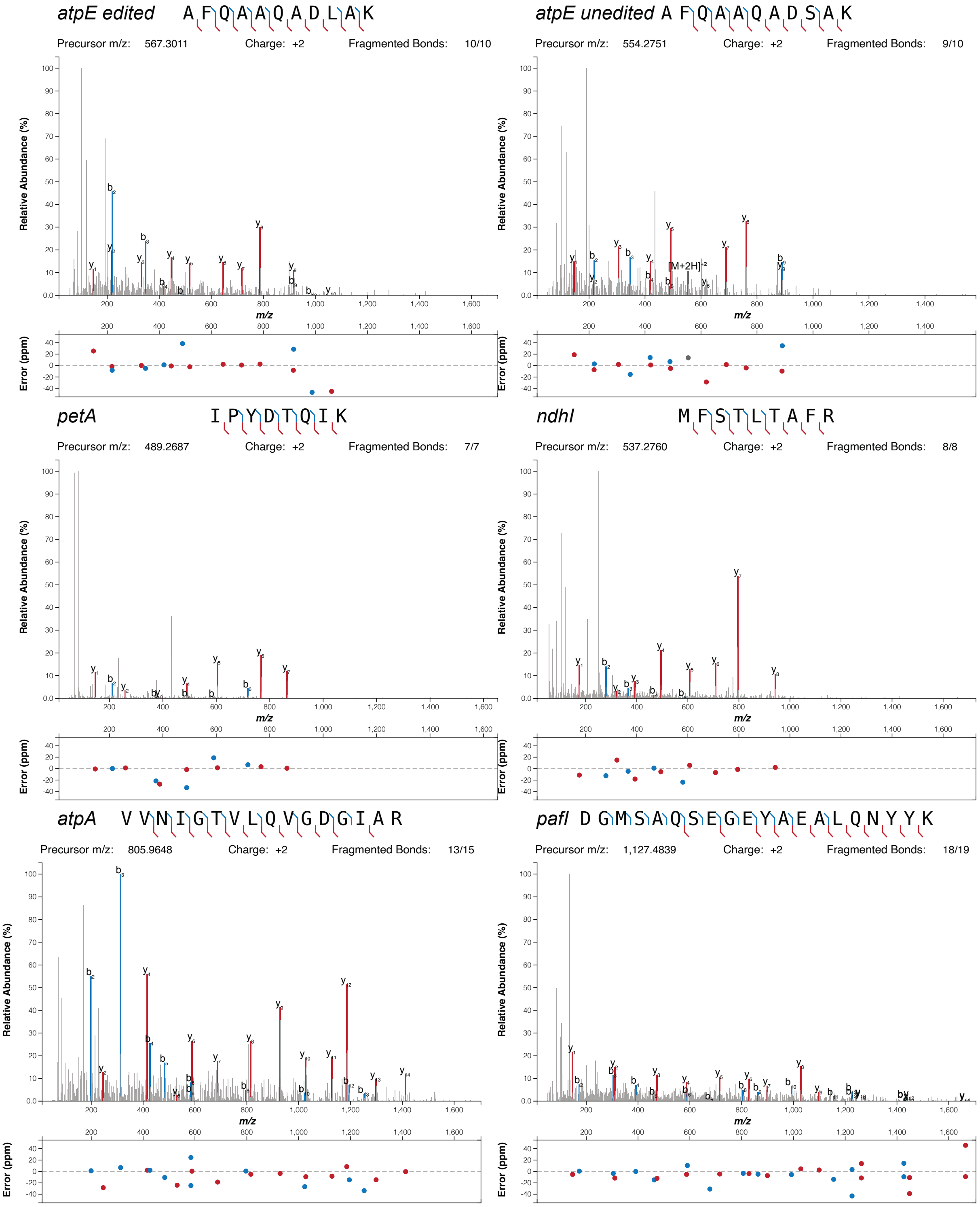

Fig. S10. Example tandem mass spectra produced from collision induced dissociation of trypsin digested thylakoid extracts. Composite plots for six peptides show the relative abundance of peptide fragments isolated from the characteristic parental mass to charge ratios (m/z) and the relative measurement error in parts per million of the daughter products.

Supplementary References

Greiner S, Lehwark P, Bock R. 2019. OrganellarGenomeDRAW (OGDRAW) version 1.3.1: expanded toolkit for the graphical visualization of organellar genomes. *Nucleic Acids Res.* 47:W59–W64.

Kuznetsov D, Tegenfeldt F, Manni M, Seppey M, Berkeley M, Kriventseva EV, Zdobnov EM. 2023. OrthoDB v11: annotation of orthologs in the widest sampling of organismal diversity. *Nucleic Acids Res.* 51:D445–D451.

Manni M, Berkeley MR, Seppey M, Simão FA, Zdobnov EM. 2021. BUSCO update: Novel and streamlined workflows along with broader and deeper phylogenetic coverage for scoring of eukaryotic, prokaryotic, and viral genomes. *Mol. Biol. Evol.* 38:4647–4654.

Simão FA, Waterhouse RM, Ioannidis P, Kriventseva EV, Zdobnov EM. 2015. BUSCO: assessing genome assembly and annotation completeness with single-copy orthologs. *Bioinformatics* 31:3210–3212.
