# Supplementary material for: High Conservation of Translation-Enabling RNA Editing Sites in Hyper-editing Ferns Implies They Are Not Selectively Neutral
Source: Mol Biol Evol. 2025 Sep 30;42(10):msaf241. doi: 10.1093/molbev/msaf241 (PMC12548569; doi:10.1093/molbev/msaf241)
Supplement: msaf241_Supplementary_Data [file msaf241_supplementary_data.zip › Supplementary_Table_Legends.pdf]

Supplementary table legends

Table S1. Mitochondrial gene complements of the eusporangiate and leptosporangiate ferns. Mitochondrial gene complements of *Psilotum nudum* (Genbank accessions KX171638-KX171639) and *Ophioglossum californicum* (Genbank accession KX171637), *Haplopteris ensiformis* (Genbank accessions OM867545-OM867553), and the *Salviniales* ferns *Azolla filiculoides* (Genbank accessions MN400566-MN400574), *Azolla rubra* (PQ554530-554666), *Azolla pinnata* (PQ554567-PQ554603), *Salvinia molesta* (PQ554641-554677), *Marsilea mutica* (PQ554604-554640).

Table S2. Summary of RNA editing events in the chloroplast genome and in mitochondrial genes of *Salviniales* ferns.

Table S3. Maximum parsimony estimates of RNA editing site gains and losses in Salviniales chloroplasts and mitochondria

Table S4. N/S ratios of editable C and U sites in *Salviniales* chloroplasts and mitochondria.

Table S5. RNA editing sites within protein-coding genes of the *Salviniales* ferns. Chloroplast and mitochondrial genes were aligned and sites were named according to the relative position of the alignment. These sites were used for comparative analysis and generation of profile likelihood plots.

Table S6. Log-likelihoods of RNA editing site partitions. Log-likelihoods for different evolutionary models based on alternative partitions of editing sites into different classes. In each case a single-partition model is compared to a two-partition model and the goodness of fit of the two models compared by a likelihood ratio test, with the p-value calculated assuming the test statistic follows a $\chi 2$ distribution with one degree of freedom.

Table S7. Editing per protein-coding gene in *Salviniales* chloroplast and mitochondria.

Table S8. Co-occurence of start codon creation and other RNA editing sites in *Marsilea mutica*. One tailed likelihood test of start codon editing sites in *Marsilea mutica* chloroplast vs. sites within 150 nucleotides (in the same Illumina read) show correlation of paired editing sites. Many transcripts containing an edited start codon contain unedited internal codon editing sites.
