# Supplementary material for: High Conservation of Translation-Enabling RNA Editing Sites in Hyper-editing Ferns Implies They Are Not Selectively Neutral
Source: Mol Biol Evol. 2025 Sep 30;42(10):msaf241. doi: 10.1093/molbev/msaf241 (PMC12548569; doi:10.1093/molbev/msaf241)
Supplement: msaf241_Supplementary_Data [file msaf241_supplementary_data.zip › Supporting Information Legend.pdf]

Supporting information

SI1. Updated annotation for the *Azolla filiculoides* chloroplast genome (MF177094). An updated annotation file is provided in .gb format. This updated annotation file includes annotations for the *ycf94* gene, the *ffs* ncRNA, modification of start coordinates for *psbC*, *rps11* and *atpI*, and modification of stop coordinates for *rpl20*, *petD* and *rpoA*. RNA editing site annotations in this file are sites detected using the RNA editing detection pipeline described in this manuscript.
